# Synthesis and Physicochemical Characterization of the Impurities of Pemetrexed Disodium, an Anticancer Drug

**DOI:** 10.3390/molecules200610004

**Published:** 2015-05-29

**Authors:** Olga Michalak, Mariusz M. Gruza, Anna Witkowska, Iwona Bujak, Piotr Cmoch

**Affiliations:** 1 Pharmaceutical Research Institute, 8 Rydygiera Street, 01-793 Warsaw, Poland; E-Mails: m.gruza@ifarm.eu (M.M.G.); a.witkowska@ifarm.eu (A.W.); i.bujak@ifarm.eu (I.B.); 2 Institute of Organic Chemistry, Polish Academy of Sciences, 44/52 Kasprzaka Street, 01-224 Warsaw, Poland; E-Mail: piotr.cmoch@icho.edu.pl

**Keywords:** pemetrexed, impurities, NMR, diastereoisomers

## Abstract

A physicochemical characterization of the process-related impurities associated with the synthesis of pemetrexed disodium was performed. The possibility of pemetrexed impurities forming has been mentioned in literature, but no study on their structure has been published yet. This paper describes the development of the synthesis methods for these compounds and discusses their structure elucidation on the basis of two-dimensional NMR experiments and MS data. The identification of these impurities should be useful for the quality control during the production of the pemetrexed disodium salt.

## 1. Introduction

**Figure 1:**
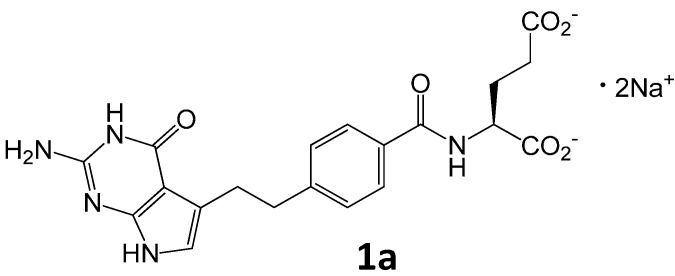
Chemical structures of pemetrexed as the disodium salt **1a**.

Pemetrexed (**1a**, Figure 1) is an antifolate antineoplastic agent that exerts its action by disrupting folate-dependent metabolic processes essential for cell replication. It acts by inhibiting three enzymes used in purine and pyrimidine synthesis *de novo*—thymidylate synthase (TS), dihydrofolate reductase (DHFR), and glycinamide ribonucleotide formyltransferase (GARFT) [1,2]. By inhibiting the formation of precursor purine and pyrimidine nucleotides, pemetrexed prevents the formation of DNA and RNA, which are required for the growth and survival of both normal and cancer cells.

A pharmaceutical product containing pemetrexed disodium (**1a**) as the active ingredient is used for the treatment of malignant pleural mesothelioma (MPM) in combination with cisplatin and as a second line agent for the treatment of advanced or metastatic non-small cell lung cancer (NSCLC). Currently, the drug is used as a single agent or in combination with other chemotherapeutic agents for the treatment of other types of cancer, such as breast cancer, bladder cancer, colorectal carcinoma and cervical cancer [3,4].

The product was originally developed by Taylor and co-workers [5] at Princeton University and is available on the market under the brand name ALIMTA^®^ (Lilly). It is a sterile lyophilized powder for intravenous infusion.

The U.S. Food and Drug Administration (FDA) [6] and the European Medicine Agency (EMA) [7] require complete physicochemical characteristic not only for an active pharmaceutical ingredient (API), but also for its key synthetic intermediates. In addition, the determination of a drug substance impurity profile, including known, especially pharmacopeial impurities [8], as well as other unknown impurities, can have a significant impact on the quality and safety of drug products.

The health implications of impurities can be significant because of their potential teratogenic, mutagenic or carcinogenic effects. Therefore, the International Conference on Harmonization (ICH) sets a high standard for the purity of drug substances [9]. If the dose is less than 2 g/day, impurities over 0.10% are expected to be identified, qualified and controlled. If the dose exceeds 2 g/day, the qualification threshold is lowered to 0.05%. It is therefore essential to control and monitor the impurities both in the APIs and the finished drug products. It is also a crucial issue in drug development and manufacturing.

This paper describes a study on identification, synthesis and characterization of the impurities formed during the pemetrexed disodium synthesis. The study will help to understand the formation of the impurities in the pemetrexed disodium synthesis and provide a clue on how to obtain a pure compound.

## 2. Results and Discussion

### 2.1. Synthesis of Pemetrexed Disodium

**Scheme 1:**
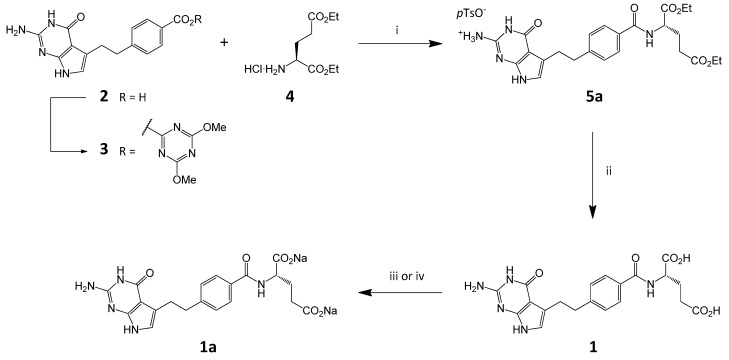
Synthesis of pemetrexed disodium. *Conditions*: (i) (a) CDMT, NMM, DMF, RT; (b) **4**; (c) *p*-TSA; (ii) (a) NaOH_aq_, (b) HCl_aq_; (iii) to **1a·7H_2_O**: (a) NaOH_aq_, (b) HCl_aq_; (iv) to **1a** (amorphic form): NaOMe, MeOH.

Convergent synthesis of pemetrexed disodium heptahydrate from key synthetic intermediates (Scheme 1) is well documented and involves firstly the preparation of the *p*-toluenesulfonic acid salt (**5a**) [10]. The acid **2** is activated for coupling by reaction with 2-chloro-4,6-dimethoxytriazine (CDMT) in presence of *N*-methylmorpholine (NMM) to form an active ester **3** and then reacted with diethyl L-glutamate **4**. The product of peptide coupling **5** is isolated as *p*-toluenesulfonate **5a** and then saponified to produce a free acid form of the drug substance (**1**). Finally, the pH is adjusted to pH 8 and the crystalline disodium salt **1a** is isolated as the heptahydrate form (**1a·7H_2_O**).

However, we have found a new method for the preparation of pemetrexed disodium **1a** in an amorphous form which involves the deprotonation of pemetrexed diacid (**1**) in the presence of sodium methoxide under anhydrous conditions [11].

During the study of process developing we observed that the product, pemetrexed disodium, contained a number of impurities; six of them were identified (Table 1).

At early stage of development process [11] we found that impurity level in some batches of pemetrexed varied in the range from 0.05% to 0.5% (HPLC). Further study was undertaken to find out if the measured impurities limits comply with the documentation requirements and if the product may be registered as the API. One of the requirements is to prove that the substance meets the characteristics described in the Pharmacopoeia, *i.a.*, the impurities limits not exceeded specified values.

For routine process controls of batches of the API as well as for development of analytical methods the possession of impurities of appropriate quantity and quality is required. Some impurity standards may be obtained commercially (at a high cost), some of them, mainly those which have not been previously described, are not available commercially. In both cases the elaboration of methods for their synthesis is very advantageous.

### 2.2. Structure of Impurities

The compounds shown in Table 1 were taken into account as potential impurities of the final pemetrexed disodium:

**Table 1:** Main impurities of pemetrexed.

Name	Structure
*N*-Methyl impurity **6**	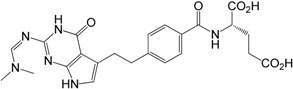
*N*,*N*-Dimethylformamidine impurity **7**	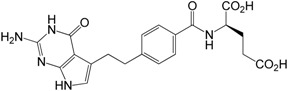
Enantiomeric impurity (***R***)-**1**	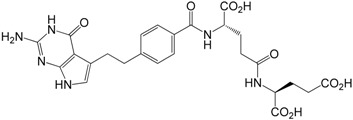
γ-Dipeptide impurity **8**	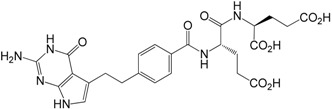
α-Dipeptide impurity **9**	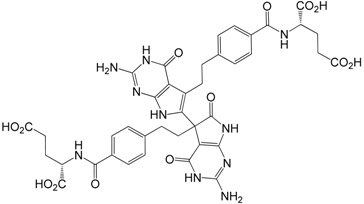
Dimer impurity **10** (diasteroisomeric mixture)	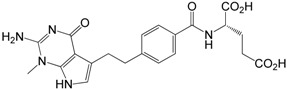

Only the impurities (***R***)-**1** (Impurity E), **6** (Impurity A), **8** (Impurity D), **10** (Impurity B and C) have been documented in the European Pharmacopoeia [8]. A detailed synthetic processes and structure confirmation have not been reported for **8**, **9**, **10**. Compounds **6** and **7** were mentioned previously, but without full characterization [12,13]. Detailed HPLC analyses for **6**, **8**, **10** were also described [14].

The impurities collected in Table 1 were synthesized and fully characterized by different techniques (IR, NMR, MS, HPLC, DSC).

### 2.3. Source and Preparation of Impurities

#### 2.3.1. *N*-Methyl Impurity **6**

The *N*-methyl impurity **6** of pemetrexed is formed while condensing the benzoic acid **2** with diethyl L-glutamate **4** in the presence of CMDT and NMM. Kjell and coworkers [12] suggest that the decomposition of the excess CDMT·NMM complex produces a methylating agent which is capable of methylating the N1-nitrogen of dezazaguanine moiety and giving **6** as the final result.

**Scheme 2:**
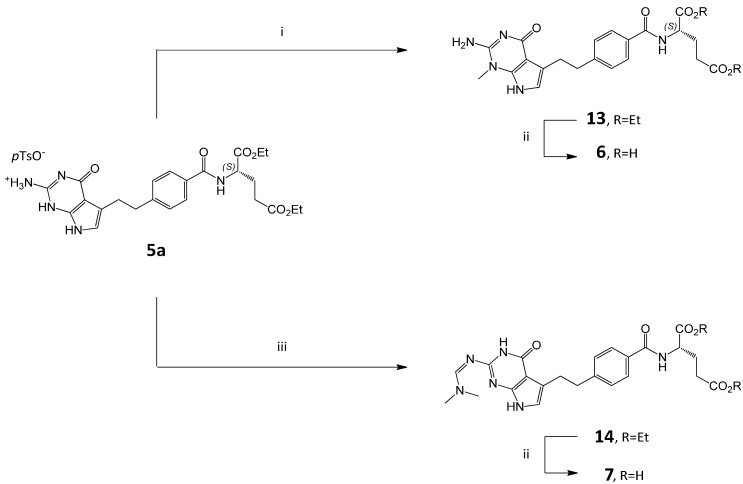
Syntheses of impurities 6 and 7. *Conditions*: (i) CH_3_I, Et_3_N, DMF; (ii) (a) NaOH_aq_, (b) HCl_aq_; (iii) DMF-DMA, *p*-TSA, DMF.

In the first route, derivative **6** was generated by alkylation of **1a** with methyl iodide in the presence of triethylamine, followed by the treatment with 1N NaOH and purification by preparative TLC chromatography on the silica gel. However, this simple attempt gave **6** with very low yield (only 5%). We have exploited a different concept for the synthesis of **6** by alkylation of diester **5a**with methyl iodide in the presence of triethylamine to *N*-methyl diester **13** and its subsequent saponification (Scheme 2).

#### 2.3.2. N,N-Dimethylformamide Impurity **7**

Recently, it has been reported [13] that when the condensation reaction between acid **2** and diethyl L-glutamate **4** is performed in the presence of *N*,*N*-dimethylformamide (DMF) as a solvent, it results in the formation **14** which subsequently after saponification results in the formation of impurity **7**.

In order to prepare derivative **14**, we adopted the procedure described in literature [13] which involved reacting **5a** with dimethylformamide-dimethylacetal (DMF-DMA) in the presence of *p-*toluenesulfonic acid (*p*-TSA) at 60 °C. We found that high excess of *p-*TSA can be decreased from 10 to 1 equivalent (coming from *p*-TSA salt **5a**). However, the addition of DMF and anhydrous conditions are required. After these modifications compound **14** was prepared in good yield and was converted to **7** by basic hydrolysis with 1N NaOH at room temperature followed by the acid treatment (Scheme 2) [15].

#### 2.3.3. Enantiomer of Pemetrexed (***R***)-**1**

Compound (***R***)**-1**, mentioned in Pharmacopeia, is a D-enantiomer of pemetrexed disodium. The presence of this impurity can be detected by a chiral HPLC method [8]. It probably arises from the trace amounts of D-enantiomer in commercial diethyl L-glutamate **4** or is formed during the hydrolysis of the ethyl esters of **1** in an alkaline medium at ambient or higher temperature (e.g., >30°) [13]. This impurity was prepared starting from diethyl D-glutamate, following a synthetic process analogous to the synthesis of pemetrexed disodium (Scheme 1). Impurity (***R***)**-1** is characterized by the same ^1^H-NMR, ^13^C-NMR and mass spectrum as pemetrexed disodium.

#### 2.3.4. γ-Dipeptide Impurity **8**

In the course of our investigation on the pemetrexed disodium synthesis, we realized that the source of impurity **8** is α-ethyl L-glutamate **11** which can be present in the starting diethyl L-glutamate **4**. We envisioned that triacid **8** could result from the condensation of monoester **11** with the acid **2**, followed by the formation of **19** through coupling α-monoester of pemetrexed **18** with **4** and the saponification of the ethyl ester groups.

Our synthesis of impurity **8** includes first the preparation of α-ethyl L-glutamate **11**. The *N*-protected α-ethyl ester **17** was isolated as the dicyclohexylammonium salt and purified by crystallization [16,17]. The decomposition of the salt with sulfuric acid and the removal of benzyloxycarbonyl group (Cbz) by catalytic hydrogenolysis finally gave **11**.

**Scheme 3:**
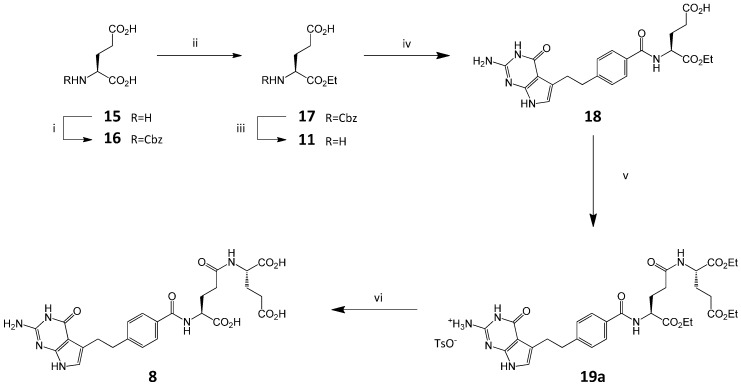
Synthesis of impurity **8**. *Conditions*: (i) NaOH, ClCO_2_Ph; (ii) (a) Et_3_N, BrEt, DMF, (b) Cy_2_NH, (c) 1M H_2_SO_4aq_; (iii) Pd/C; (iv) **2**, CDMT, NMM, DMF; (v) (a) **4**, CDMT, NMM, DMF; (b) *p*-TSA, EtOH; (vi) (a) NaOH_aq_; (b) HCl_aq_.

The conversion of **11** to **19a** was achieved through conventional peptide coupling with CDMT/NMM and the subsequent formation of the *p*-TSA salt. The final hydrolysis of the triester **19a** with 1 N NaOH followed by the acid treatment provided triacid **8** (Scheme 3).

#### 2.3.5. α-Dipeptide Impurity **9**

**Scheme 4:**
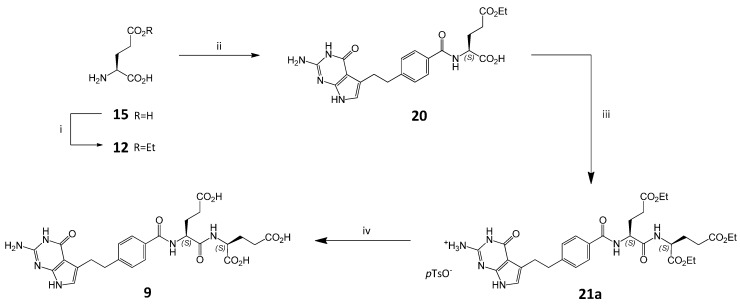
Synthesis of impurity **9**. *Conditions*: (i) HCl, EtOH; (ii) 2, CDMT, NMM, DMF; (iii) (a) 4, CDMT, NMM, DMF; (b) *p*TsOH, EtOH; (iv) (a) 1 M NaOH_aq_, (b) 1 M HCl_aq_

Similarly, if starting diethyl L-glutamate **4** contains some amount of γ-ethyl L-glutamate **12**, then during the preparation of pemetrexed disodium α-dipeptide impurity **9** may appeared. At the beginning for the synthesis of dipeptide **9** a similar synthetic procedure to that described above for the preparation of dipeptide **8** was used (Scheme 4).

The reaction of γ-ethyl ester **12** (contamination of the main compound **4**) with **2** led to γ-ethyl ester **20**, which after further condensation with **4** gave *p-*toluenesulfonate **21a**. This compound, after hydrolysis with NaOH at ambient temperature followed by acidification with HCl, gave triacid **9**.

Surprisingly, the HPLC analysis of **9** revealed the presence of two separated equivalent peaks. For compound **8** such phenomenon was not observed. LC-MS showed that the molecular weight for both peaks of **9** was the same. In the NMR spectra of **21a** and **9** we also observed doubled signals (for details, see Section 2.4).

One possible explanation of the observed data is the formation of diastereoisomeric mixture. Most probably monoester **20** (in fact the substituted benzoyl-(*S*)-α-amino acid) undergoes a racemization during the activation/coupling step with diester **4** (Scheme 4, step iii) [18], thus a mixture of diastereoisomeric triesters **21** (*S*,*S*- and *R*,*S-*configuration) is formed which after hydrolysis give the mixture of diastereoisomeric triacids **9** (*S*,*S-* configuration and *R*,*S*-configuration), respectively.

**Scheme 5:**
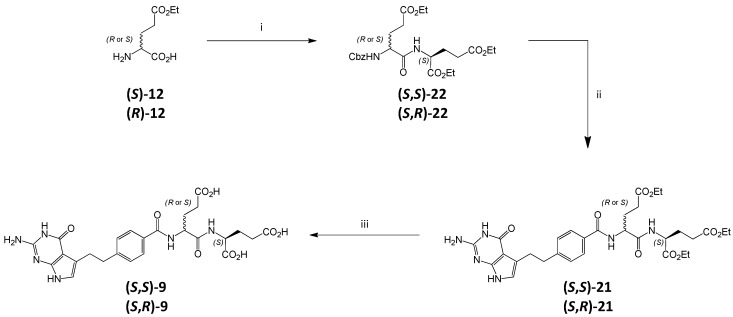
Synthesis of **(*S***,***S*)-9** and **(*S***,***R*)-9**. *Conditions*: (i) (a) CbzCl, (b) **4**, DIPEA, HATU, DMF (ii) (a) Pd/C, H_2_, EtOH; (b) **2**, HATU; (iii) (a) 1 M NaOH_aq_, (b) 1 M HCl_aq_.

A simple change in the coupling conditions to eliminate racemization in coupling **20** with amine **4** failed. Using HATU [19] instead of CDMT/NMM also resulted in the mixture of diastereoisomeric triesters **21**. In order to resolve this problem and obtain standard samples, we decided to prepare independently both diastereoisomers of impurity **9** (***S***,***S*-** and ***S***,***R*-**) employing a different synthetic route (Scheme 5).

In this approach *N*‑Cbz-protected (***S*** or ***R***) glutamic acid derivatives were used for coupling leading to dipeptides **22** (***S***,***S*-** and ***S***,***R***-) which were subsequently converted into **9** (***S***,***S*-** and ***S***,***R***-). It is well known that contrary to N^α^-acyl protected amino acids which racemize readily during the activation/coupling of the carboxyl group for the amide bond formation, in the case of the urethane-type amine protecting groups (as Cbz) the tendency of racemization is largely suppressed [20].

The γ-monoesters **12** (with *S*- or *R*- configuration, respectively) were *N*-Cbz-protected [21] and then coupled with diester **4** to obtain enantiomerically pure protected dipeptides **22** (***S***,***S*-** and ***S***,***R***-). After cleaving the Cbz-group, each of the enantiomerically pure amines was coupled with acid **2** to give both diastereoisomeric triesters **21**, which after hydrolysis led to diastereoisomeric triacids (***S***,***S***)**-9** and (***S***,***R***)**-9**, respectively.

The NMR, MS and HPLC analyses confirmed that (***S***,***S***)**-9** and (***S***,***R***)**-9** were pure single compounds (in HPLC designated by different retention times 27.0 and 26.6 min for (***S***,***S***)**-9** and (***S***,***R***)**-9**, respectively).

#### 2.3.6. Dimer Impurity **10**

Dimeric impurity **10** (as diasteroisomeric mixture) of pemetrexed might be formed during the basic hydrolysis of **5a** to the pemetrexed disodium salt. According to the *European Pharmacopoeia* [8], in order to prepare impurities **10**, pemetrexed disodium is dissolved in 0.1 M NaOH_aq_ and heated at 70 °C for 40 min. After cooling to room temperature, the mother solution was diluted with water to obtain the “reference solution”.

We have modified this procedure and developed a purification method for impurities **10**. Pemetrexed disodium was dissolved in 0.1 M NaOH_aq_ and heated under reflux for 3 days (TLC control). Then, the mixture was cooled and 10% HCl_aq_ was added to adjust pH ≈ 3. A formed precipitate was filtered and purified by chromatography to get the mixture of **10**. The structure of the investigated compound was confirmed by NMR (discussed hereinafter).

### 2.4. Structure Elucidation by Analytical Methods

The structures of all studied impurities were identified using the results of various 2D NMR spectra, including the COSY, ^1^H-^13^C/^15^N gradient selected HSQC, as well as ^1^H-^13^C/^15^N gradient selected HMBC sequences.

**Enantiomeric impurity** (***R***)**-1**: compound (***R***)-**1** in a diacid form as well as the disodium salt are the enantiomers of the main compound **1**. The comparison of the multinuclear NMR spectra recorded for (***R***)-**1** with those registered currently and published earlier for (***S***)-**1** [5,22] undoubtedly confirm the structure of the enantiomeric (***R***)-**1** (Table 2).

***N*-Methyl impurity 6**: The analysis of the results of different NMR spectra, especially ^1^H-^15^N HMBC experiment, strongly supports the proposed structure **6**. The most significant effect was observed for the ^15^N-NMR chemical shifts. Unfortunately, compound **6** (acid form) forms a gel in the DMSO solution and that is why we used NMR data of diethyl ester **13** for comparison. In the ^1^H-^15^N *g*-HMBC spectrum of **13** a strong correlation peak was noticed between the protons of CH_3_ group at δ = 3.56 ppm and nitrogen at δ = −274.2 ppm. This “cross-peak” identifies the position of the methylation which is an N3 nitrogen atom. Additionally, in the ^1^H-^13^C HMBC experiment CH_3_ protons (introduced onto nitrogen atom) correlate with two carbons (δ = 152.1 (C2) and 139.3 ppm (C4)) and as a consequence strongly support the place of the methylation.

Introducing a methyl group onto the N3 nitrogen atom causes a strong shielding increase noticeable for N3 and C4 nuclei by *ca*. 66 and 12 ppm, respectively. Similar shielding effects at C4 are visible in the case of *p*-toluenosulfonic salts (***R***)**-5a**, **21a** and **19a**, (Experimental), which explains the protonation site of the neutral compounds at the N3 atom. The NMR data for **6**/**13** are given in Table 2.

***N*,*N*-Dimethylformamidine impurity 7**: The sets of the ^1^H/^13^C-NMR chemical shifts (whose assignment comes from the analysis of the results of more advanced 2D experiments, including ^1^H-^13^C HSQC/HMBC and ^1^H-^15^N HSQC/HMBC correlations) unambiguously confirm the presence of an imine part in the molecule of the obtained compound and thereby the structure of impurity **7**. The most important and significant effect was observed for the nitrogen nucleus at the C2 atom. The ^15^N shielding decrease of *ca.* 130 ppm between the exocyclic nitrogen at C2 in the structures of **1** and **7** is responsible for the exchange of the nitrogen atom character from the amino to imine group. The comparison of ^1^H/^13^C- and ^15^N-NMR data for **1** with that obtained for **7** (change of the NH_2_ group to N=CH-N(CH_3_)_2_), for which the ^1^H-NMR spectra are described in literature [13], leads to the observation of a few shielding/deshielding effects on the atoms in close neighborhood of the replacement. The most important one is carbon C2 deshielded by *ca*. 3 ppm when compared with its position in **1**. Moreover, a quite surprising deshielding effect is noticeable at the N1/H1 pair (Table 2). The H1 proton is deshielded by *ca*. 0.6 ppm, whereas in the case of nitrogen N1 the same effect is stronger by *ca.* 13 ppm. Other more distant atoms also experience deshielding effects. For carbons C5, C8 and nitrogen N9 these changes are *ca*. 2 ppm, 1.5 ppm and 1.5 ppm, respectively (Table 2).

**Table 2:** Comparison of the NMR data for compounds **1**, **6**, **7**, **10** and **13** with the correlations observed in the HSQC and HMBC spectra.

Pos. ^a^	1		13 ^b^		6 ^b^		7 ^b^		10		Pos. ^a^
	^1^H (ppm)	^13^C/^15^N (ppm)	^1^H (ppm)	^13^C/^15^N (ppm)	^1^H (ppm)	^13^C ^c^ (ppm)	^1^H (ppm)	^13^C/^15^N (ppm)	^1^H (ppm)	^13^C/^15^N (ppm)	
**1**	10.18	−235.7	–	^d^	–	–	10.78	−222.3	10.09; 10.60	−236.2; ^d^	**1; 1′**
**2**	–	152.2	–	152.1	–	152.0	–	155.6	–	150.3; 152.1 or 157.6	**2; 2′**
**C2-NH_2_**	6.03	−310.8	^d^	^d^	^d^	–	–	−176.1	6.02; 6.90	−311; ^d^	**C2-NH_2_ C2′-NH_2_**
**3**	–	−207.6		−274.2	–	–	–	NR	–	^d^	**3; 3′**
**4**	–	151.3	–	139.3	–	139.0	–	150.1	–	152.1 or 157.6; 164.0	**4; 4′**
**5**	–	98.8	–	100.3	–	100.9	–	101.7	–	99.5; 92.71	**5; 5′**
**6**	–	159.4	–	163.3	–	163.8	–	160.2	–	159.1; 157.8	**6; 6′**
**7**	–	117.7	–	119.7	–	119.4	–	117.8	–	114.2; 51.7	**7; 7′**
**8**	6.32	113.5	6.64	114.9	6.42	113.2	6.47	115.0	–	123.0; 179.6	**8; 8′**
**9**	10.62	−241.2	–	−243.6	–	–	10.82	−239.8	10.74; 10.87	−238.9; −233.7	**9; 9′**
**10**	2.87	28.0	2.95	27.1	2.94	26.8	2.92	27.9	2.64; 2.57	28.1; 34.2	**10; 10′**
**11**	2.99	36.2	3.00	35.8	2.99	35.6	3.01	36.2	2.67; 2.45	37.8; 29.8	**11; 11′**
**12**	–	146.2	–	145.8	–	145.5 ^e^	–	146.2	–	146.5; 145.0	**12; 12′**
**13**	7.30	128.2	7.32	128.2	7.28	127.7	7.31	128.2	7.26; 7.30	128.0; 128.1	**13; 13′**
**14**	7.80	127.4	7.81	127.5	7.75	126.8	7.80	127.4	7.80	127.4 + 127.5	**14 + 14′**
**15**	–	131.3	–	131.2	–	131.5 ^e^	–	131.3	–	131.4 + 131.5	**15 + 15′**
**16**	–	166.6	–	166.6	–	165.9	–	166.5	–	166.4 + 166.6	**16 + 16′**
**17**	8.52	−265.9	8.65	−266.8	8.12	–	8.51	−265.9	2 × 8.52	2 × −265.9	**17 + 17′**
**18**	4.41	52.0	4.44	52.0	4.39	52.1	4.41	51.9	4.41	51.9	**18 + 18′**
**19**	1.97; 2.11	26.0	2.02; 2.12	25.7	2.00; 2.08	26.4	1.96; 2.10	26.0	1.97; 2.10	26.0	**19 + 19′**
**20**	2.37	30.4	2.44	30.2	2.34	30.6	2.36	30.5	2.32-2.40	30.4	**20 + 20′**
**C21-OH**	^d^	174.0	–	172.2	^d^	173.5	12.40	173.9	–/12.39	173.9 + 173.9	**C21 + 21′-OH**
**C22-OH**	^d^	173.5	–	171.8	^d^	173.1	12.40	173.5	–/12.39	173.5 + 173.5	**C22 + 22′-OH**

^a^ Positions of atoms are showed on Figure 2; ^b^ Signals for substituents (^1^H/^13^C/^15^N): **13**: 4.11/60.5 and 4.05/59.9 (2 × –CH_2_CH_3_); 3.56/32.4 (N3–CH_3_); 1.19 and 1.17/14.1 (2 × –CH_2_CH_3_); **6**: 3.49/31.5 (N3–CH_3_); **7**: 8.49/156.9–N=CHN(CH_3_)_2_); −281.2 –N=CHN(CH_3_)_2_); 3.01/34.5 and 3.11/40.5 (–N=CHN(CH_3_)_2_); ^c^ registered at 353 K; ^d^ signals not observed; ^e^ Kjell *et al.* [12] described signal assignments for C12 and C15 in reversed order.

**Figure 2:**
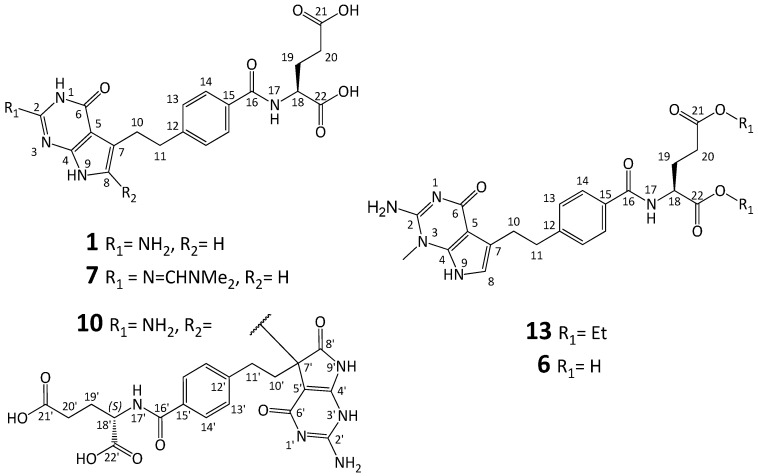
Numbering of atoms in the NMR spectra description for compounds **1**, **6**, **7**, **10** and **13** (Table 2).

**γ-Dipeptide impurity 8 and α-dipeptide impurities 9**:

The NMR spectra and HPLC analysis of compounds **8** and **9** showed some unexpected results. As mentioned before, in the case of impurity **8** the HPLC method revealed one compound, whereas for impurity **9** two peaks in HPLC were observed. A detailed analysis of the 2D-NMR experiments for **8** taken in the DMSO solution leads to the ^1^H/^13^C- and ^15^N-NMR chemical shift assignment and complete confirmation of the structure of this impurity (Table 3).

The same detailed analysis of the 2D-NMR data was performed for **9**, confirming the structure of this impurity, although a doubled set of ^1^H/^13^C-NMR and even ^15^N-NMR signals was noticed there.

At the beginning the observation of the doubled ^1^H/^13^C-NMR signals forced us to check if we were not dealing with rotamers. A well-known method employing ^1^H-NMR spectroscopy [23] was useless, because the ^1^H-NMR signals overlapped. We decided to raise the temperature and observe the ^13^C-NMR spectrum. However, the temperature increase (between 25–100 °C or 298–373 K) resulted in no significant changes in the ^1^H/^13^C-NMR spectra and based on these experiments we ruled out the hypothesis of the rotamers presence.

Another possible explanation of the observed data was the formation of the diastereoisomeric mixture during the synthesis. To prove this hypothesis, both diastereoisomers of impurity **9** (***S***,***S*-** and ***S***,***R*-**) were independently synthesized employing a substantially racemization-free synthetic route based on the use of the *N*‑Cbz protecting group described in Section 2.3.5 (Scheme 5).

The NMR, MS and HPLC analysis of the synthesized compounds confirmed that (***S***,***S***)**-9** and (***S***,***R***)**-9** were pure single diastereoisomers characterized by a single set of the ^1^H/^13^C- and ^15^N-NMR signals. The ^1^H/^13^C-NMR spectra recorded for both diastereoisomeric compounds (***S***,***S***)**-9** and (***S***,***R***)**-9** were very similar to each other, with minor differences in the narrow ranges of the ^1^H/^13^C-NMR chemical shifts (Table 3). These are visible for the C16-C28 chain and especially for the H17-H20 and H23-H26 protons.

**Table 3:** NMR Data for compounds **8**, (***S***,***S***)**-9** and (***S***,***R***)**-9** with the correlations observed in the HSQC and HMBC spectra.

	8			(*S*,*S*)-9			(*S*,*R*)-9	
Pos. ^a^	^1^H (ppm)	^13^C/^15^N (ppm)	Pos. ^a^	^1^H (ppm)	^13^C/^15^N (ppm)	Pos. ^a^	^1^H (ppm)	^13^C (ppm)
**1**	10.18	−236.4	**1**	10.17	−236.3	**1**	10.15	–
**2**	–	152.2	**2**	–	152.3	**2**	–	152.2
**C2-NH_2_**	6.03	−311.2	**C2-NH_2_**	6.01	−311.1	**C2-NH_2_**	6.01	–
**3**	–	−208.0	**3**	–	−208.0	**3**	–	–
**4**	–	151.3^p^	**4**	–	151.4	**4**	–	151.3
**5**	–	98.8	**5**	–	98.8	**5**	–	98.7
**6**	–	159.3	**6**	–	159.5	**6**	–	159.3
**7**	–	117.6	**7**	–	117.5	**7**	–	117.7
**8**	6.31	113.5	**8**	6.31	113.6	**8**	6.32	113.4
**9**	10.61	−241.4	**9**	10.60	−241.4	**9**	10.60	–
**10**	2.86	28.0	**10**	2.85	28.1	**10**	2.86	28.0
**11**	2.96	36.1	**11**	2.97	36.2	**11**	2.98	36.1
**12**	–	146.1	**12**	–	146.2	**12**	–	146.1
**13**	7.29	128.2	**13**	7.28	128.2	**13**	7.28	128.1
**14**	7.80	127.4	**14**	7.78	127.5	**14**	7.79	127.5
**15**	–	131.4	**15**	–	131.5	**15**	–	131.5
**16**	–	166.4	**16**	–	166.6	**16**	–	166.4
**17**	8.57	−265.6	**17**	8.34	−265.5	**17**	8.30	–
**18**	4.35	52.3	**18**	4.46	52.8	**18**	4.50 (1H, m)	52.8
**19**	1.94 + 2.10	26.5	**19**	1.92 + 2.03	27.1	**19**	1.94 + 2.01	27.2
**20**	2.28	31.8	**20**	2.34	30.5	**20**	2.32	30.5
**21**	–	171.8	**C21-OH**	^c^	174.3	**C21-OH**	^c^	174.1
**C22-OH**	12.40 ^b^	173.5	**22**	–	171.7	**22**	–	171.6
**23**	8.15	−260.0	**23**	8.21	−263.2	**23**	8.20	–
**24**	4.21	51.2	**24**	4.22	51.3	**24**	4.23	51.1
**25**	1.75 + 1.93	26.4	**25**	1.81 + 1.99	26.3	**25**	1.79 + 1.99	26.4
**26**	2.27	30.1	**26**	2.30	30.1	**26**	2.25	29.9
**C27-OH**	12.40 ^b^	173.8	**C27-OH**	^c^	173.9	**C27-OH**	^c^	173.7
**C28-OH**	12.40 ^b^	173.4	**C28-OH**	^c^	173.3	**C28-OH**	^c^	173.1

^a^ Positions of atoms are showed on Figure 3; ^b^ probably one common signal for the exchanging protons of carboxylic groups; ^c^ signals not observed.

**Figure 3:**
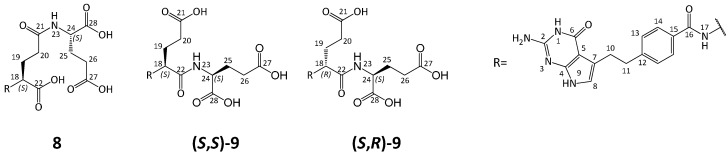
Numbering of atoms in the NMR spectra description for compounds **8**, (***S***,***S***)**-9** and (***S***,***R***)**-9** (Table 3).

All the results strongly support the hypothesis that during the synthesis of impurity **9** according to Scheme 4, the coupling of **20** with **4** leads to the diastereomeric mixture of **22**.

**Diasteroisomeric dimer impurity 10**:This impurity was mentioned in Pharmacopeia but has not been reported in literature. The HR mass spectrum of **10** showed the exact mass *m*/*z* = 867.2709, perfectly corresponding with the Pharmacopeia structure. Yet more convincing proof of the structure of **10** comes from the analysis of various NMR experiments. The ^1^H/^13^C spectra for this impurity in DMSO showed double sets of signals which may be considered as an existence of two structurally identical fragments. This observation and further analysis of the long-range ^1^H-^13^C and ^1^H-^15^N *g*-HMBC correlations leading to ^1^H/^13^C-and ^15^N-NMR signals assignment, indicates that both “pemetrexed” parts in this structure are connected with each other via the C7/C8 bond. 

One part of this dimeric molecule looks identically as the pemetrexed molecule (both C7 δ = 114.2 ppm and C8 δ = 123.0 ppm carbons are aromatic), whereas in the second part carbons C7 and C8 change their character in such a way that C7 (δ = 51.7 ppm) becomes more aliphatic, while the C8 nucleus (δ = 179.6 ppm) is much more deshielded than when they are positioned in the “pemetrexed” part (Table 2). The above mentioned effects are as follows: the shielding increase of *ca*. 62 ppm and at the same time the shielding decrease of *ca*. 55 ppm for C7 and C8, respectively.

## 3. Experimental Section

### 3.1. General Information

The starting 4-[2-(2-amino-4-oxo-4,7-dihydro-1*H*-pyrrolo[2,3-*d*]pyrimidin-5-yl)ethyl]benzoic acid (**2**) was obtained from Neorganic, Warsaw, Poland. γ-Ethyl L- and D-glutamates were prepared according to the literature procedure [21]. Other materials, solvents and reagents were of commercial origin and used without additional operations. All reactions were carried out in ambient temperature, if not stated otherwise. Coupling reactions were performed in anhydrous solvents.

The purity of the examined compounds was determined using HPLC/UHPLC methods with the chromatography system UltiMate™ 3000RS UHPLC (Dionex Corporation, Sunnyvale, CA, USA) equipped with an autosampler and a DAD 3000RS detector.

Method A: Gemini C18 column (150 mm × 4.6 mm, 3 µm; Phenomenex, Torrance, CA, USA) was placed in a thermostated column heater at 25 °C. The mobile phases consisting of A (4 g/L dipotassium hydrogen phosphate; pH 5.2) and B (acetonitrile) were used with the gradient mode at the flow rate of 0.9 mL/min. The samples were prepared at a concentration of about 0.5 mg/mL and were diluted in 0.4 g/L of dipotassium hydrogen phosphate. The injection volume was 10 µL. The UV detection at 230 nm was used.

The TLC separations were performed on the TLC silica gel 60 F254 on alumina sheets (Merck and/or Sigma-Aldrich). The visualization was performed by UV light (254 and/or 365 nm) and [24].

The specific rotation [α]_D_ was calculated from an optical rotation measurement performed on the Perkin Elmer 341 Polarimeter (PerkinElmer, Waltham, MA, USA) at the wavelength of 589 nm (D line of a sodium lamp), at 20 °C.

The melting points were determined by differential scanning calorimetry (DSC) carried out by means of the DSC822 with an IntraCooler (Mettler Toledo GmbH, Schwerzenbach, Switzerland).

The ^1^H-NMR (600 MHz), ^13^C-NMR (150 MHz) and 15N-NMR (60 MHz) spectra recorded in the DMSO-*d*_6_ solutions with the Varian-NMR-vnmrs600 spectrometer (Varian Inc. Palo Alto, CA, USA) at 298 K temperature, equipped with a 600 MHz PFG Auto XID (^1^H/^15^N-^31^P 5 mm) indirect probehead. To identify the structures of pemetrexed impurities correctly, careful analysis of the results of 1D and 2D NMR experiments was employed. The 1D and 2D measurements covered: ^1^H selective NOESY, 2D: COSY, the ^1^H-^13^C gradient selected HSQC and HMBC optimized for ^1^*J*(C-H) = 150 Hz and ^n^*J*(C-H) = 8 Hz, respectively. The ^15^N NMR chemical shifts were obtained on the basis of the 2D ^1^H-^15^N gradient selected HSQC and HMBC experiments, optimized for ^1^*J*(N-H) = 90 Hz and ^n^*J*(N-H) = 6 Hz, respectively. Standard experimental conditions and standard Varian programs (ChemPack 4.1) were used. The ^1^H- and ^13^C-NMR chemical shifts are given relative to the TMS signal at 0.0 ppm, whereas neat nitromethane at 0.0 ppm was used as a standard for the ^15^N-NMR chemical shifts. The concentration of the solutions used for the measurements was about 20–30 mg of the compounds in 0.6 cm^3^ of deuterated DMSO (DMSO-*d*_6_). Used abbreviations: s—singlet, d—doublet, t—triplet, m—multiplet, ov—overlapped signals. The integrals are not presented due to the signals overlapping in most cases. Some ^1^H-NMR chemical shifts for all the compounds studied are given as the averaged value of the center of multiplets read from the ^1^H-^13^C g-HSQC experiments.

The mass spectra were recorded on the MaldiSYNAPT G2-S HDMS (Waters Co., Milford, MA, USA) Spectrometer via electrospray ionization (ESI-MS).

### 3.2. Synthesis of Impurity **6**

#### 3.2.1. (2*S*)-2-[[4-[2-(2-Amino-1-methyl-4-oxo-4,7-dihydro-1*H*-pyrrolo[2,3-*d*]pyrimidin-5-yl)ethyl]benzoyl]amino]pentanedioic Acid Diethyl Ester (**13**)

To the solution of **5a** (6.0 g, 9.16 mmol) in DMF (40 mL) triethylamine was added (3.20 mL, 22.96 mmol), followed by methyl iodide (2.06 mL, 33.09 mmol) and the solution was left at room temperature for 72 h. Then CH_2_Cl_2_ (80 mL) and water (80 mL) were added. The layers were separated and the aqueous layer was extracted with CH_2_Cl_2_ (1 × 40 mL).The combined organic layers were dried over anhydrous MgSO_4_ and concentrated. The residue was dissolved in MeOH (10 mL) and ^i^Pr_2_O (50 mL) was added. The resulted precipitate was filtered, washed with ^i^Pr_2_O (2 × 10 mL), and dried to give **13** (4.0 g, 88%).

TLC: *R*_F_ = 0.18 (CHCl_3_/MeOH/Et_3_N 8:2:1)

^1^H-NMR: δ: 8.65 (1H, d, *J* = 7.5 Hz, N17-H), 7.81 (2H, d, *J* = 8.0 Hz, H14), 7.32 (2H, d, *J* = 8.0 Hz, H13), 6.64 (1H, s, H8), 4.44 (1H, m, H18), 4.11 (2H, m, -CH_2_CH_3_ at C22), 4.05 (2H, q, *J* = 7.1 Hz, -CH_2_CH_3_ at C21), 3.56 (3H, s, CH_3_ group at N3), 3.00 (2H, m, H11), 2.95 (2H, m, H10), 2.44 (2H, m, H20), 2.12 and 2.02 (2H, 2 × m, both H19 protons), 1.19 and 1.17 (2 × 3H, 2 × q, *J* = 7.1 Hz, both CH_3_ groups of -CH_2_CH_3_ at C22 and C21);

^13^C-NMR: δ: 172.18 (CO, C21), 171.82 (CO, C22), 166.58 (CO, C16), 163.26 (probably C6), 152.11 (probably C2), 145.81 (C12), 139.31 (probably C4), 131.19 (C15), 128.17 (C13), 127.46 (C14), 119.69 (C7), 114.89 (C8), 100.31 (C5), 60.53 (-CH_2_CH_3_ at C22), 59.92 (-CH_2_CH_3_ at C21), 51.95 (C18), 35.79 (C11), 32.45 (CH_3_ group at N3), 30.17 (C20), 27.13 (C10), 25.69 (C19), 14.1 (2 × CH_3_ groups of -CH_2_CH_3_ at C22 and C21);

^15^N-NMR δ: −274.2 (N3), −266.8 (N17), −243.6 (N9), the ^15^N-NMR signals of NH_2_ at C2 and N1 not recorded in the ^1^H-^15^N *g*-HSQC/HMBC experiments.

HRMS: calcd for C_25_H_32_N_5_O_6_ *m*/*z* = 498.2353, found *m*/*z* = 498.2346.

#### 3.2.2. (2*S*)-2-[[4-[2-(2-Amino-1-methyl-4-oxo-4,7-dihydro-1*H*-pyrrolo[2,3-*d*]pyrimidin-5-yl)ethyl]benzoyl]amino]pentanedioic Acid (**6**)

Compound **13** (3.50 g, 7.03 mmol) was treated with 1M NaOH_aq_ (30 mL) and stirred at *RT* for 2 h. The reaction mixture was diluted with EtOH (30 mL) and water (30 mL) and adjusted to pH 3.0 with 1 M HCl. The resulting slurry was heated to 60–65 °C and then cooled to *RT*. The solid was filtered, washed with EtOH (2 × 10 mL) and dried *in vacuo* at 40 °C for 24 h. Crude **6** was purified by flash chromatography on a silica gel column using CH_2_Cl_2_/MeOH/H_2_O/NH_3_ as the mobile phase (40:40:5:2 *v*/*v*). The respective fractions were collected and concentrated. The residue was dissolved in water (50 mL) and the pH was adjusted to 2–3 with 1 M HCl. EtOH (220 mL) was added and stirred for 30 min. The suspension was filtered and the solid was washed with EtOH/H_2_O (20 mL) and dried at 40 °C to obtain **6** (2.51 g, 81%, HPLC purity 99.05%).

TLC: *R*_F_ = 0.44, (CHCl_3_/MeOH/NH_3_, 2:2:1).

Mp. 270 °C;

${\left[\text{α}\right]}_{\text{D}}^{20}$ = +10.03 (c = 1, DMSO);

^1^H-NMR (353 K) δ: 8.12 (1H, s, N17-H), 7.75 (2H, m, H14), 7.28 (2H, m, H13), 6.42 (1H, s, H8), 4.39 (1H, m, H18), 3.49 (3H, s, CH_3_ at N3), 2.99 (2H, m, H11), 2.94 (2H, m, H10), 2.34 (2H, m, H20), 2.08 and 2.00 (2H, m, H19);

^13^C-NMR (353 K) δ: 173.54 (CO, C21), 173.06 (CO, C22), 165.85 (CO, C16), 163.75 (C6), 152.04 (C2), 145.47 (C12), 139.03 (C4), 131.48 (C15), 127.72 (C13), 126.75 (C14), 119.43 (C7), 113.18 (C8), 100.94 (C5), 52.06 (C18), 35.63 (C11), 31.53 (N3-CH_3_), 30.59 (C20), 26.77 (C10), 26.41 (C19);

HRMS: calcd for C_21_H_24_N_5_O_6_ *m*/*z* = 442.1727, found *m*/*z* = 442.1724.

FT-IR: [cm^−1^] 3226–3127 (N-H_ν_, O-H_ν_); 2926 (C-H_ν_); 1682–1640 (C=O_ν_, C=N_ν_); 1612; 1504 (C=C_ν_); 1543 (N-H_δ_, C=N_ν_); 1450–1402 (C-H_δ_, C-N_ν_); 1236 (C-O_ν_); 697 (C-H_γ_, N-H_γ_).

### 3.3. Synthesis of Impurity **7**

#### 3.3.1. (2*S*)-2-[[4-[2-(2-(Dimethylamino)methyleneamino-4-oxo-4,7-dihydro-1*H*-pyrrolo[2,3-*d*]pyrimidin-5-yl)ethyl]benzoyl]amino]pentanedioic Acid Diethyl Ester (**14**)

Compound **5a** (6.5 g, 9.9 mmol) was suspended in *N*,*N*-dimethylformamide dimethyl acetal (DMF-DMA, 56 mL) and anhydrous DMF (18 mL) and stirred at *RT* for 3 days (TLC control). The reaction mixture was poured into water (100 mL) and extracted with CH_2_Cl_2_ (3 × 30 mL). The combined organic layers were dried over anhydrous Na_2_SO_4_ overnight, then filtered and concentrated to a thick liquid mass. Water (100 mL) was added to the mass and stirred (the use of a mechanical stirrer or a rotary evaporator is recommended) for 4 h at *RT*. The solid precipitate was filtered off and washed with water (25 mL), then dried. The solid was dissolved in MeOH (30 mL) in 60–65 °C and ^i^Pr_2_O (250 mL) was slowly added (over 45–60 min), then the mixture was cooled and stirred for 1 h at *RT*. The obtained precipitate was filtered and washed with ^i^Pr_2_O (2 × 15 mL) and dried under vacuum at 40 °C to afford **14** (4.37 g, 81.5%).

TLC: *R*_F_ = 0.78 (CHCl_3_/MeOH, 8:2);

Mp. 193 °C;

^1^H-NMR δ: 10.82 (1H, s, N9-H), 10.77 (1H, s, N1-H), 8.63 (1H, d, *J* = 7.4 Hz), 8.49 (1H, s, -N=CH-N(CH_3_)_2_), 7.79 (2H, m, H14), 7.31 (2H, m, H13), 6.46 (1H, s, H8), 4.43 (1H, m, H18), 4.11 (2H, m, CH_2_ of -CH_2_CH_3_ at C22), 4.05 (2H, q, *J* = 7.1 Hz, CH_2_ of -CH_2_CH_3_ at C21), 3.12 (3H, s, one of the CH_3_ group of -N=CH-N(CH_3_)_2_), 3.01 (5H, m, one of the CH_3_ group of -N=CH-N(CH_3_)_2_) and both H11), 2.92 (2H, m, both H10), 2.44 (2H, m, both H20), 2.11 and 2.01 (2H, 2 × m, both H19), 1.19 (3H, s, CH_3_ of -CH_2_CH_3_ at C22) and 1.17 (3H, s, CH_3_ of -CH_2_CH_3_ at C21);

^13^C-NMR δ: 172.20 (CO, C21), 171.82 (CO, C22), 166.62 (CO, C16), 160.11 (probably C4), 156.91 (-N=CH-N(CH_3_)_2_), 155.52 (C2), 150.06 (probably C6), 146.28 (C12), 131.10 (C15), 128.18 (C13), 127.39 (C14), 117.73 (C7), 114.93 (C8), 101.64 (C5), 60.51 (CH_2_ of -O-CH_2_CH_3_ at C22), 59.90 (CH_2_ of -CH_2_CH_3_ at C21), 51.95 (C18), 40.44 (one of CH_3_ group of -N=CH-N(CH_3_)_2_), 36.17 (C11), 34.43 (one of CH_3_ group of -N=CH-N(CH_3_)_2_), 30.17 (C20), 27.89 (C10), 25.70 (C19), 14.05 (both CH_3_ groups of -CH_2_CH_3_ at C22 and C21);

^15^N-NMR δ: −281.3 (-N=CH-N(CH_3_)_2_), −266.9 (N17), −240.0 (N9), −175.8 (-N=CH-N(CH_3_)_2_. N1 and N3 not recorded in the ^1^H-^15^N *g*-HMBC experiment;

HRMS: calcd for C_27_H_35_N_6_O_6_ *m*/*z* = 539.2618, found *m*/*z* = 539.2610.

#### 3.3.2. (2*S*)-2-[[4-[2-(2-(Dimethylamino)methyleneamino-4-oxo-4,7-dihydro-1*H*-pyrrolo[2,3-*d*]pyrimidin-5-yl)ethyl]benzoyl]amino]pentanedioic Acid (7)

Compound **14** (0.77 g, 1.43 mmol) was treated with NaOH_aq_ (0.25 g in 11 mL H_2_O), the mixture was cooled to 0–5 °C and stirred for 15 min. EtOH (11 mL) was added to the solution and stirred for 1 h at 0–5 °C. The pH was adjusted to 3 with 1N HCI, then EtOH was evaporated. The suspension was filtered and the solid was washed with EtOH:H_2_O (1:1, 1 × 5 mL) and dried at 40 °C to obtain **7** (0.41 g, 70.0%, HPLC purity 80.0%).

TLC: *R*_F_ = 0.49 (CHCl_3_/MeOH, 1:2);

NMR data are shown in Table 2.

^1^H-NMR (600 MHz, DMSO): 12.4 (2H, broad s, probably protons of both COOH groups), 10.82 (1H, d, *J* = 1.8 Hz, N9-H), 10.78 (1H, s, N1-H), 8.51 (1H, d, *J* = 7.7 Hz, N17-H), 8.49 (1H, s, -N=CH-N(CH_3_)_2_), 7.80 (2H, m, H14), 7.31 (2H, m, H13), 6.47 (1H, d, *J* = 1.8 Hz, H8), 4.41 (1H, m, H18), 3.11 (3H, s, one of the CH_3_ groups of -N=CH-N(CH_3_)_2_), 3.01 (5H, m, one of the CH_3_ groups of -N=CH-N(CH_3_)_2_ and both protons at C11), 2.92 (2H, m, H10), 2.36 (2H, t, *J* = 7.5 Hz, C20), 2.10 (1H, m, one of the protons at C19), 1.96 (1H, m, one of the protons at C19);

^13^C-NMR (150 MHz, DMSO): 173.92 (CO, C21), 173.50 (CO, C22), 166.52 (CO, C16), 160.16 (probably C6), 156.94 (-N=CH-N(CH_3_)_2_), 155.55 (C2), 150.10 (probably C4), 146.17 (C12), 131.34 (C15), 128.20 (C13), 127.38 (C14), 117.76 (C7), 115.00 (C8), 101.67 (C5), 51.91 (C18), 40.47 (one of the CH_3_ groups of -N=CH-N(CH_3_)_2_), 36.20 (C11), 34.47 (one of the CH_3_ groups of -N=CH-N(CH_3_)_2_), 30.45 (C20), 27.94 (C10), 25.95 (C19);

^15^N-NMR: −281.2 (-N=CH-N(CH_3_)_2_), −265.9 (N17), −239.8 (N9), −222.3 (N1), −176.1 (-N=CH-N(CH_3_)_2_), N3 not recorded in ^1^H-^15^N *g*-HSQC/HMBC experiments;

HRMS: calcd for C_23_H_27_N_6_O_6_ *m*/*z* = 483.1992, found *m*/*z* = 483.1985.

FT-IR: ν [cm^−1^] 3320–3126(N-H_ν_, O-H_ν_); 2922(C-H_ν_); 1683 (C=O_ν_); 1635 (C=N_ν_); 1532 (N-H_δ_, C=N_ν_); 1505 (C=C_ν_); 1420 (C-H_δ_); 1354 (C-N_ν_); 1120 (C-O_ν_); 840 (C-H_γ_).

Adjusting the reaction mixture’s pH to 8 enabled the isolation of trisodium salt **7a** (details are given in the Supplementary Part).

### 3.4. Synthesis of Impurity (**R**)***-1***

#### 3.4.1. (2*R*)-2-[[4-[2-(2-Amino-4-oxo-4,7-dihydro-1*H*-pyrrolo[2,3-*d*]pyrimidin-5-yl)ethyl]benzoyl]amino]-pentanedioic Acid Diethyl Ester, *p*-TSA Salt ((***R***)**-5a**)

*N*-methylmorpholine (NMM, 10.6 mL, 96.41 mmol) was added to the suspension of **2** (10 g, 33.56 mmol) in DMF (16 mL) and CH_2_Cl_2_ (95 mL), followed by 2-chloro-4,6-dimethoxy-1,3,5-triazine (CDMT, 6.48 g, 36.90 mmol), and the resulting solution was stirred at 38–40 °C for 2 h. To this solution diethyl D-glutamate hydrochloride (7.88 g, 32.87 mmol) was added and the resulting mixture was stirred for 2 h. Then water (100 mL) was added and the mixture was stirred for 15 min. The organic layer was separated and the aqueous phase extracted with CH_2_Cl_2_ (1 × 70 mL). The organic layers were collected, washed with 1 M NaHCO_3aq_ (1 × 70 mL), and concentrated under reduced pressure to afford oil.

EtOH (180 mL) was added to the oil, followed by the solution of the *p*-toluenesulfonic acid monohydrate in EtOH (15.96 g in 180 mL) and the resulting suspension was heated under reflux for 2 h. The mixture was cooled to RT, the crystals of (***R***)**-5a** were filtered and washed with EtOH (2 × 60 mL). The wet cake was reslurried in EtOH (400 mL), refluxed for 1 h and cooled to *RT*. The crystals were filtered, washed with EtOH (2 × 60 mL) and dried *in vacuo* at 40 °C for 24 h to provide **(*R*)-5a** (14.48 g, 66%).

TLC: *R*_F_ = 0.51 (CHCl_3_/MeOH 8:2);

${\left[\text{α}\right]}_{\text{D}}^{20}$ = +0.79 (c = 1, DMSO);

Mp. 268 °C;

NMR data are identical as for the L-enantiomer (***S***)**-5a**;

HRMS: calcd for C_24_H_30_N_5_O_6_ *m*/*z* = 484.2196, found *m*/*z* = 484.2187.

#### 3.4.2. (2*R*)-2-[[4-[2-(2-Amino-4-oxo-4,7-dihydro-1*H*-pyrrolo[2,3-*d*]pyrimidin-5-yl)ethyl]benzoyl]amino]-pentanedioic Acid ((***R***)**-1**)

Compound (***R***)**-5a** (14.4 g, 21.98 mmol) was treated with 1 M NaOH_aq_(112 mL), the mixture was stirred at room temperature. After 1 h the reaction mixture was adjusted to pH 8.0 with 1N HCl_aq_ and heated to 55–60 °C. EtOH (560 mL) was added to the solution. After cooling to *RT*, the precipitated solid was collected by filtration and washed with EtOH (2 × 80 mL). The wet solid (12.84 g) was dissolved in water (120 mL) and the solution was heated to 55–60 °C. EtOH (500 mL) was added and then the mixture cooled to *RT*. The solid was filtered, washed with EtOH (2 × 80 mL) and dried in vacuo at 35 °C for 48 h to provide (***R***)**-1** (9.8 g, 87%, 99.4% total HPLC purity, 99.9% chiral HPLC purity).

Mp. 240 °C;

${\left[\text{α}\right]}_{\text{D}}^{20}$ = −18.5 (c = 1, H_2_O);

NMR data are identical as for L-enantiomer (***S***)**-1**;

HRMS: calcd for C_20_H_21_N_5_O_6_Na *m*/*z* = 450.1390, found *m*/*z* = 450.1405;

### 3.5. Synthesis of Impurity ***8***

#### 3.5.1. *N*-Benzyloxycarbonyl-L-glutamic Acid (**16**)

*N*-Benzyloxycarbonyl-L-glutamic acid was prepared according to literature [21]. After recrystallization from ethyl acetate/hexanes (1:6), a white solid was obtained.

TLC *R*_F_ = 0.48 (CHCl_3_/MeOH/NH_3_ 2:2:1);

${\left[\text{α}\right]}_{\text{D}}^{20}$ = −9.0 (c = 2, AcOH);

Mp. 118 °C;

^1^H-NMR (600 MHz, DMSO): 12.4 (2H, very broad s, both protons of -COOH); 7.57 (1H, d, *J* = 8.1 Hz, NH), 7.35 and 7.29 (5H, m, Ph), 5.03 (2H, s, CH_2_ of Cbz), 3.99 (1H, m, CH-NHCbz), 2.29 (2H, m, both H of CH_2_β), 1.96 and 1.75 (2H, 2m, both H of CH_2_α);

^13^C-NMR (150 MHz, DMSO): 173.77 (COγ), 173.62 (COα), 156.20 (CO of NHCbz), 137.00, 128.4, 127.8, 127.7 (Ph), 65.47 (CH_2_ of Cbz), 53.08 (CH-NHCbz), 30.12 (CH_2_β), 26.13 (CH_2_α);

HRMS calcd for C_13_H_15_NO_6_Na *m*/*z* = 304.0797, found *m*/*z* = 304.0799.

#### 3.5.2. *N*-Benzyloxycarbonyl-L-glutamic Acid α-Ethyl Ester (**17**)

The dicyclohexylammonium (DCHA) salt of compound **16** was prepared according to literature [25].

TLC *R*_F_ = 0.75 (CHCl_3_/MeOH/NH_3_ 2:2:1); DCHA salt

Mp. 160 °C (lit. [25] 159–160 °C);

${\left[\text{α}\right]}_{\text{D}}^{20}$ = −12.2 (c=0.5, MeOH) (lit. [25] [α]23−25 D = −11.7 (c = 2, MeOH));

^1^H-NMR δ: 8.12 (1H, d, *J* = 6.6 Hz, NH), 7.44–7.25 (5H, m, all protons of Ph ring), 5.02 (2H, 2×d, *J* = 12.7 Hz, CH_2_ of Cbz), 4.09 (2H, CH_2_ of -O-CH_2_CH_3_), 4.01 (1H, m, CH-NH), 2.73, 2.15, 1.92–1.80, 1.75, 1.67, 1.56, 1.28–1.05;

^13^C-NMR δ: 174.69, 172.37, 156.05, 137.03, 128.32, 127.78, 127.66, 65.35, 60.32, 53.91, 51.94, 32.08, 31.17, 26.64, 25.41, 24.35, 14.06.

1 M H_2_SO_4 aq_ (100 mL) was added to the suspension of DCHA salt of **16** (8.63 g, 17.58 mmol) in AcOEt (200 mL). After 10 min water (100 mL) was added to the reaction mixture, the organic layer was separated and the aqueous phase extracted with AcOEt (2 × 150 mL). The organic layers were collected, washed with water (2 × 50 mL), dried over anhydrous MgSO_4_ and concentrated under reduced pressure to afford **17** (5.0 g, 92%).

${\left[\text{α}\right]}_{\text{D}}^{20}$ = −20.7 (c = 0.5, MeOH);

^1^H-NMR δ: 7.76 (1H, d, *J* = 7.7 Hz, NH), 7.38–7.29 (5H, m, all protons of Ph ring), 5.03 (2H, 2×d, *J* = 12.5 Hz, CH_2_ of Cbz), 4.08 (2H, m, CH_2_ of -CH_2_CH_3_), 4.04 (1H, m, CH-NH), 2.28 (2H, m, -CH_2_-COOH), 1.93 (1H, m), 1.76 (1H, m), 1.17 (3H, t, *J* = 7.1Hz, CH_3_);

^13^C-NMR δ: 173.96, 172.12, 156.11, 136.94, 128.34, 127.83, 127.70, 65.46, 60.50, 53.27, 30.23, 26.09, 14.03;

HRMS calcd for C_15_H_19_NO_6_Na *m*/*z* = 332.1110, found *m*/*z* = 332.1114.

#### 3.5.3. L-Glutamic Acid α-ethyl Ester (**11**)

The solution of **17** (5.0 g, 16.2 mmol) in EtOH (150 mL) was hydrogenated in the presence 10% Pd/C (0.4 g) for 2.5 h. After filtration through a Celite pad, the solution was evaporated and dropped into Et_2_O (50 mL). After cooling in the refrigerator, the white precipitate was filtered, washed with Et_2_O, and dried in vacuum at 30 °C to give **11** (2.6 g, 93%).

TLC: *R*_F_ = 0.65 (CHCl_3_/MeOH/NH_3_ 2:2:1);

${\left[\text{α}\right]}_{\text{D}}^{20}$ = +27.0 (c = 2, 1 M HCl);

Mp. 90 °C;

^1^H-NMR δ: 5.00 (3H, very broad s, -NH_2_ and -COOH), 4.10 (2H, m, ‑CH_2_CH_3_), 3.37 (1H, m, -CHNH_2_), 2.29 (2H, m, both H of CH_2_β), 1.83 and 1.64 (2H, 2m, both H of CH2α), 1.20 (3H, t, *J* = 7.0 Hz, -CH_2_CH_3_);

^13^C-NMR δ: 174.80 (COα), 174.26 (COγ), 60.20 (-CH_2_CH_3_), 53.12 (-CHNH_2_), 30.84 (CH_2_β), 29.18 (CH_2_α), 14.07 (-CH_2_CH_3_);

^15^N-NMR δ: −351.5;

HRMS: calcd for C_7_H_14_NO_4_ *m*/*z* = 176.0923, found *m*/*z* = 176.0926.

#### 3.5.4. (2*S*)-2-[[4-[2-(2-Amino-4-oxo-4,7-dihydro-1*H*-pyrrolo[2,3-*d*]pyrimidin-5-yl)ethyl]benzoyl]amino]-pentanedioic Acid 1-Ethyl Ester (**18**)

NMM (1.99 mL, 18.11 mmol), followed by CDMT (1.94 g, 11.06 mmol) were added to the suspension of acid **2** (3 g, 10.06 mmol) in DMF (30 mL) and the resulting solution was stirred at *RT* for 2 h. α-ethyl L-glutamate **11** (1.85 g, 10.56 mmol) was added to this solution and the resulting mixture was stirred for 24 h. The reaction mixture was poured into water (30 mL) and extracted with CH_2_Cl_2_ (3 × 30 mL). The organic phases were collected, dried over anhydrous MgSO_4_ and concentrated to afford **18** (2.83 g, 61%).

TLC: *R*_F_ = 0.67_._ (CHCl_3_/MeOH, 4:6);

^1^H-NMR δ: 11.1 (1H, broad s, probably N1-H), 10.57 (1H, s, N9-H), 10.38 (1H, s, N17-H), 7.80 (2H, m, H14), 7.23(2H, m, H13), 6.49 (2H, s, NH_2_ at C2), 6.27 (1H, s, H8), 4.20 (1H, m, H18), 4.07 (2H, m, -CH_2_CH_3_ at C22), 2.92 (2H, m, H11), 2.82 (2H, m, H10), 2.19 (2H, m, H20), 1.95 (2H, m, H19), 1.16 (3H, t, *J* = 7.1 Hz, -CH_2_CH_3_ at C22);

^13^C-NMR δ: 176.78 (CO, C21), 172.28 (CO, C22), 165.96 (C16), 159.70 (C6), 152.79 (C2), 151.52 (C4), 145.93 (C12), 131.16 (C15), 128.16 (C13), 127.30 (C14), 117.69 (C7), 113.09 (C8), 98.66 (C5), 60.09 (-O-CH_2_CH_3_), 54.12 (C18), 36.16 (C11), 33.97 (C20), 28.00 (C10), 26.37 (C19), 14.15 (-O-CH_2_CH_3_);

^15^N-NMR δ: −309.9 (NH_2_ at C2), −263.7 (N17), −241.8 (N9); N1 and N3 not recorded in ^1^H-^15^N *g*-HMBC experiment;

HRMS calcd for C_22_H_25_N_5_O_6_Na *m*/*z* = 478.1703, found *m*/*z* = 478.1694.

#### 3.5.5. (2*S*)-2-[[(4*S*)-4-[[4-[2-Amino-4-oxo-4,7-dihydro-1*H*-pyrrolo[2,3-*d*]pyrimidin-5-yl)ethyl]benzoyl]amino]-4-(ethoxycarbonyl)butanoyl]amino]pentanedioic Acid Diethyl Ester, *p*-TSA Salt (**19a**)

NMM (1.57 mL, 14.28 mmol) was added to the suspension of **18** (2.33 g, 5.12 mmol) in DMF (20 mL), followed by CDMT (1.08 g, 6.15 mmol), and the resulting solution was stirred at *RT* for 3 h. diethyl L-glutamate hydrochloride **4** (1.34 g, 5.61 mmol) was added to this solution and the resulting mixture was stirred for 24 h. Then water (30 mL) and CH_2_Cl_2_ (30 mL) were added to the reaction mixture which was stirred for 15 min. The organic layer was separated and the aqueous phase extracted with CH_2_Cl_2_ (2 × 25 mL). The organic layers were collected, washed with 1 M NaHCO_3aq_ (1 × 35 mL), and concentrated under reduced pressure to afford oil.

EtOH (40 mL), followed by the solution of *p-*TSA·H_2_O in EtOH (2.46 g in 40 mL EtOH) were added to the oil and the resulting suspension was heated under reflux for 2 h. Then the mixture was cooled to *RT*, the crystals were filtered, washed with EtOH (2 × 15 mL) and dried *in vacuo* at 40 °C for 24 h to provide **19a** (1.05 g, 25%).

TLC: *R*_F_ = 0.46 (CHCl_3_/MeOH 8:2);

Mp. 232 °C;

^1^H-NMR δ: 11.39 (1H, s, probably N1-H), 11.26 (1H, s, N9-H), 8.67 (1H, d, *J* = 7.3 Hz, N-H17), 8.27 (1H, d, *J* = 7.6 Hz, N-H23), 7.80 (2H, m, H14), 7.53 (2H, d, *J* = 8.0 Hz, *p*-TSA), 7.30 (2H, m, H13), 7.15 (2H, d, *J* = 8.0 Hz, *p*-TSA), 6.51 (1H, s, H8), 4.39 (1H, m, H18), 4.24 (1H, m, H24), 4.11 (2H, q, *J* = 7.0 Hz, CH_2_ of -CH_2_CH_3_ at C22), 4.07 (2H, q, *J* = 7.0 Hz, CH_2_ of -CH_2_CH_3_ at C27), 4.04 (2H, q, *J* = 7.0 Hz, CH_2_ of -CH_2_CH_3_ at C26), 2.97 (2H, m, H11), 2.90 (2H, m, H10), 2.36 (2H, m, H26), 2.34–2.26 (5H, m, H20 and CH_3_ of *p*-TSA), 2.09 (1H, m, one of H19 protons), 1.96 (2H, m, one of the H19 protons and one of H25 protons), 1.81 (1H, m, one of the H25 protons);

^13^C-NMR δ: 172.10 (CO, C27), 172.00 (CO, C22), 171.74 (2×CO, C28 and C21), 166.47 (CO, C16), 157.45 (probably C6), 150.72 (probably C3), 145.70 (C12), 144.94 (C_IV_, *p*-TSA), 139.81 (probably C4), 138.12 (C_IV_, *p*-TSA), 131.32 (C15), 128.16 (C13), 128.16 (CH, p-TSA), 125.50 (CH, *p*-TSA), 118.92 (C7), 115.13 (C8), 99.00 (C5), 60.48 (-O-CH_2_CH_3_ at C27), 60.44 (-CH_2_CH_3_ at C22), 59.90 (-CH_2_CH_3_ at C26), 52.38 (C18), 51.22 (C24), 35.74 (C11), 31.38 (C20), 29.84 (C26), 27.25 (C10), 26.19 (C19), 26.03 (C25), 20.77 (CH_3_ group of p-TSA), 14.09, 14.05 and 14.00 (carbons of CH_3_ of -O-CH_2_CH_3_ groups);

^15^N-NMR δ: −266.8 (N23), −266.4 (N17), −242.1 (N9);

HRMS: calcd for C_31_H_41_N_6_O_9_ *m*/*z* = 641.2935, found *m*/*z* = 641.2930.

#### 3.5.6. (2*S*)-2-[[(4*S*)-4-[[4-[2-Amino-4-oxo-4,7-dihydro-1*H*-pyrrolo[2,3-*d*]pyrimidin-5-yl)ethyl]benzoyl]amino]-4-carboxybutanoyl]amino]pentanedioic Acid (**8**)

Compound **19a** (1.86 g, 2.28 mmol) was treated with 1 M NaOH_aq_ (15 mL), the mixture was stirred at *RT*. After 1 h the reaction mixture was adjusted to pH 3.0 with 1N HCl_aq_, and the precipitate was formed. After 30 min the precipitate was filtered off, washed with water (2 × 10 mL) and dried to give crude **8**. The crude material (1.60 g) was purified by column chromatography (SiO_2_, CHCl_3_-MeOH-H_2_O-25% NH_3aq_ 40:40:5:2) to give fraction of **8**, 668 mg (HPLC 98.6%). The obtained material (660 mg) was then dissolved in water (10 mL) and the solution was acidified to pH = 3 with 10% HCl_aq_ (*ca.* 2.5 mL), forming a precipitate. The mixture was stirred at *RT* for 30 min, then the precipitate was filtered off, washed with water (3 × 15 mL) and dried to give **8** (in triacid form, 0.48 g, 37.6% from **19a**, HPLC 98.0%). A second fraction of **8** (390 mg, HPLC 91.5%) was obtained by chromatography and acidified as described above to give **8** in the triacid form (0.27 g, yield 21.4% from **19a**, HPLC purity 97.6% ).

${\left[\text{α}\right]}_{\text{D}}^{20}$ = −3.87 (c = 1, DMSO);

Mp. 155 °C;

^1^H-NMR δ: 12.40 (*ca.* 3H, broad s, 3×COOH), 10.61 (1H, s, N9-H), 10.18 (1H, s, N1-H), 8.57 (1H, d, *J* = 7.6 Hz, N17-H), 8.13 (1H, d, *J* = 7.8 Hz, N23-H), 7.80 (2H, m, H14), 7.29 (2H, m, H13), 6.31 (1H, s, H8), 6.03 (2H, s, NH_2_ at C2), 4.35 (1H, m, H18), 4.21 (1H, m, C24), 2.98 (2H, m, H11), 2.86 (2H, m, H10), 2.28 (2H, m, H20), 2.27 (2H, m, H26), 2.10 (1H, m, H19), 1.94 (2H, m, H19 and H25), 1.75 (H25);

^13^C-NMR δ: 173.77 (CO, C27), 173.54 (CO, C22), 173.39 (CO, C28), 171.75 (CO, C21), 166.43 (CO, C16), 159.30 (CO, C6), 152.22 (C2), 151.33 (probably C4), 146.12 (C12), 131.39 (C15), 128.15 (C13), 127.38 (C14), 117.64 (C7), 113.46 (C8), 98.75 (C5), 52.31 (C18), 51.19 (C24), 36.14 (C11), 31.76 (C20), 30.14 (C26), 28.02 (C10), 26.48 (C19), 26.39 (C25);

^15^N-NMR δ: −311.2 (C2-NH_2_), −265.6 (N17), −260.0 (N23), −241.4 (N9), −236.4 (N1), −208.0 (N3);

HRMS: calcd for C_25_H_27_N_6_O_9_ *m*/*z* = 555.1840, found *m*/*z* = 555.1851.

FT-IR: [cm^−1^] 3312 (N-H_ν_, O-H_ν_); 2926 (C-H_ν_); 1702 (C=O_ν_); 1647 (C=O_ν_, C=N_ν_); 1504 (C=C_ν_); 1541 (N-H_δ_, C=N_ν_); 1448, 1407 (C-H_δ_); 1330 (C-N_ν_); 1223 (C-O_ν_, C-N_ν_); 666 (C-H_γ_, N-H_γ_).

Adjusting the reaction mixture’s pH to 8 enabled the isolation of the trisodium salt **8a** (details are given in the Supplementary Part).

### 3.6. Synthesis of Impurity (**S**,**S**)***-9*** and Diastereoisomer (**S**,**R**)***-9***

#### 3.6.1. (2*S*)-2-[(2*S*)-2-Carboxybenzylamino-4-(ethoxycarbonyl)butanoyl]aminopentanedioic Acid Diethyl Ester ((***S***,***S***)**-22**)

DIPEA (3.0 mL, 17.2 mmol) was added to the suspension of *N*-Cbz protected **(*S*)-12** (1.4 g, 4.53 mmol prepared according to the literature procedure [26]) in DMF (15 mL), followed by HATU (2.07 g, 5.43 mmol). The resulting solution was stirred at *RT* for 1 h. Then diethyl L-glutamate hydrochloride **4** (1.63 g, 6.79 mmol) was added to the solution and the resulting mixture was stirred for 24 h. The reaction mixture was diluted with water (70 mL) and extracted with AcOEt (3 × 25 mL). The combined organic layers were washed with 1 M HCl_aq_ (1 × 25 mL), 5% NaHCO_3 aq_ (1 × 25 mL), sat. brine (1 × 25 mL), dried over Na_2_SO_4_, and concentrated to give crude (***S***,***S***)**-22** as oil. The oil was crystallized from the AcOEt/hexanes mixture to obtain pure dipeptide (***S***,***S***)**-22** as a solid (53.2%).

TLC: *R*_F_ = 0.52 (hexanes/AcOEt 1:1);

${\left[\text{α}\right]}_{\text{D}}^{20}$ = −20.70 (c = 1.0 MeOH);

Mp. Phase trans.: peak endothermic. 1 = 84.3 °C; peak exothermic. = 89.2 °C; peak endothermic. 2 = 109.8 °C;

^1^H-NMR δ: 8.34 (1H, d, *J* = 7.8 Hz, NH), 7.46 (1H, d, *J* = 7.8 Hz, Cbz-NH), 7.38–7.28 (5H, Ph), 5.02 (2H, dd, *J* = 12.5 Hz, CH_2_ of Cbz group), 4.26 (1H, m, CH_α_), 4.10–4.02 (7H, m, all CH_2_ of ester -CH_2_CH_3_ groups and CH_α_), 2.40–2.32 (4H, m, all CH_γ_ protons), 2.00+1.84 (2H, 2 × m, both CH_β_ protons), 1.91 + 1.78 (2H, 2 × m, both CH_β_ protons), 1.17 (9H, three overlapping triplets, all CH_3_ of ester -CH_2_CH_3_ groups);

^13^C-NMR δ: 172.3, 172.1, 171.4 (CO of ester groups), 171.6 (CO of amide group), 155.8 (CO of Cbz group), 137.0 (C_IV_ of Ph), 128.3, 127.8 and 127.6 (Ph), 65.4 (CH_2_ of Cbz group), 60.6, 59.9 and 59.8 (CH_2_ carbons of ester -CH_2_CH_3_ groups), 53.6 (CH_α_), 51.2 (CH_α_) 30.0, 29.8 (both CH_γ_ carbons), 27.2, 25.8 (both CH_β_ carbons), 14.0 and 13.9 (all CH_3_ carbons of ester -CH_2_CH_3_ groups).

HRMS calcd for [M + H]^+^ C_24_H_35_N_2_O_9_ *m*/*z* = 495.2343, found *m*/*z* = 495.2344.

#### 3.6.2. (2*S*)-2-[(2*R*)-2-Carboxybenzylamino-4-(ethoxycarbonyl)butanoyl]aminopentanedioic Acid Diethyl Ester ((***S***,***R***)**-22**)

(***R***)**-12** prepared according to the literature procedure [26] was coupled with **4** analogically to the preparation of (***S***,***S***)**-22** to give triester (***S***,***R***)**-22**.

TLC: *R*_F_ = 0.44 (hexanes/AcOEt 3:2);

${\left[\text{α}\right]}_{\text{D}}^{20}$ = −5.12 (c = 1.0 MeOH);

Mp. 90.3 °C;

^1^H-NMR δ: 8.34 (1H, d, *J* = 7.8 Hz, NH), 7.40 (1H, d, *J* = 7.8 Hz, Cbz-NH), 7.38–7.28 (5H, Ph), 5.02 (2H, m, CH_2_ of Cbz group), 4.21 (1H, m, CH_α_), 4.10–4.02 (7H, m, all CH_2_ of ester -CH_2_CH_3_ groups and CH_α_), 2.36–2.28 (4H, m, all CH_γ_ protons), 2.00 + 1.84 (2H, 2 × m, both CH_β_ protons), 1.88 + 1.78 (2H, 2 × m, both CH_β_ protons), 1.17 (9H, three overlapping triplets, all CH_3_ of ester -CH_2_CH_3_ groups);

^13^C-NMR δ: 172.4, 172.3, 171.7 (CO of ester groups), 171.5 (CO of amide group), 156.0 (CO of Cbz group), 137.0 (C_IV_ of Ph), 128.4, 127.9 and 127.7 (Ph), 65.6 (CH_2_ of Cbz group), 60.8, 60.1 and 60.0 (CH_2_ carbons of ester -CH_2_CH_3_ groups), 53.9 (CH_α_), 51.3 (CH_α_) 30.1, 29.8 (both CH_γ_ carbons), 27.4, 26.0 (both CH_β_ carbons), 14.1 and 14.0 (all CH_3_ carbons of ester -CH_2_CH_3_ groups).

HRMS calcd for [M + Na]^+^ C_24_H_34_N_2_O_9_Na *m*/*z* = 517.2162, found *m*/*z* = 517.2154.

#### 3.6.3. (2*S*)-2-[[(2*S*)-2-[[4-[2-Amino-4-oxo-4,7-dihydro-1*H*-pyrrolo[2,3-*d*]pyrimidin-5-yl)ethyl]benzoyl]amino]-4-(ethoxycarbonyl)butanoyl]amino]pentanedioic Acid Diethyl Ester ((***S***,***S***)**-21**)

The solution of triester (***S***,***S***)**-22** (800 mg, 1.62 mmol) in EtOH (20 mL) was hydrogenated in the presence of 10% Pd/C (0.4 g) for 2.5 h. After filtrating through a Celite pad, the solution was evaporated to obtain amino-triester as oil (574 mg).

Acid **2** (296 mg, 0.99 mmol) was dissolved in DMF (10 mL), then DIPEA (0.52 mL, 2.97 mmol) was added followed by HATU (498 mg, 1.29 mmol) and the resulting solution was stirred at *RT* for 30 min. Then the solution of the previously obtained amino-triester (500 mg, 1.39 mmol) in 10 mL DMF was added and the resulting mixture was stirred overnight.

The reaction mixture was diluted with water (25 mL) and extracted with AcOEt (3 × 25 mL). The combined organic layers were washed with 5% NaHCO_3 aq_ (1 × 25 mL), dried over anhydrous Na_2_SO_4_ and concentrated to give crude (***S***,***S***)**-21** as oil. The oil was crystallized from the AcOEt/MTBE mixture to obtain pure dipeptide (***S***,***S*)-21** as a solid (596 mg, 65% from (***S***,***S***)**-22**).

TLC: *R*_F_ = 0.35 (CH_2_Cl_2_/MeOH 10:0.8);

${\left[\text{α}\right]}_{\text{D}}^{20}$ = −1.20 (c = 1.0 MeOH);

Mp. 149.1 °C;

^1^H-NMR δ: 10.60 (1H, d, *J* = 2.0 Hz, H9); 10.15 (1H,s, H1); 8.37 (1H, d, *J* = 7.5 Hz, H23); 8.35 (1H, d, *J* = 7.8 Hz, H17); 7.79 and 7.28 (2×2H, AA′BB′, H13 and H14); 6.30 (1H, m, H8); 6.00 (2H, s, C2-NH_2_); 4.45 (1H, m, H18); 4.27 (1H, ddd, *J* = 5.3 Hz, *J* = 7.5Hz, *J* = 9.3 Hz, H24); 4.10–4.00 (6H, ov, m, 3 × -CH_2_CH_3_); 2.97 (2H, m, H11); 2.85 (2H, m, H10); 2.41 (2H, m, ov, H20); 2.39 (2H, m, ov, H26); 2.05 (1H, m, ov, H19); 2.01 (1H, m, ov, H25); 1.95 (1H, m, ov, H19); 1.86 (1H, m, H25); 1.18–1.14 (9H, 3 t, ov, *J* = 7.1 Hz, 3 × -CH_2_CH_3_);

^13^C-NMR δ: 172.4 (C21); 172.2 (C27); 171.6 (C22); 171.5 (C28); 166.4 (C16); 159.3 (C6); 152.2 (C2); 151.3 (C4); 146.1 (C12); 131.4 (C15); 128.1 (C13); 127.4 (C14); 117.6 (C7); 113.4 (C8); 98.8 (C5); 60.6 (CH_2_CH_3_); 59.9 (CH_2_CH_3_); 59.8 (CH_2_CH_3_); 52.5 (C18); 51.2 (C24); 36.1 (C11); 30.3 (C20); 29.8 (C26); 28.0 (C10); 26.9 (C19); 25.8 (C25); 14.04 (2C, -CH_2_CH_3_); 13.9 (-CH_2_CH_3_);

^15^N-NMR δ: −208.0 (N3); −236.3 (N1); −241.4 (N9); −263.7 (N23); −265.9 (N17); −311.2 (C2-NH_2_);

HRMS calcd for [M+H]^+^ C_31_H_41_N_6_O_9_ *m*/*z* = 641.2935, found *m*/*z* = 641.2930.

#### 3.6.4. (2*S*)-2-[[(2*R*)-2-[[4-[2-Amino-4-oxo-4,7-dihydro-1*H*-pyrrolo[2,3-*d*]pyrimidin-5-yl)ethyl]benzoyl]amino]-4-(ethoxycarbonyl)butanoyl]amino]pentanedioic Acid Diethyl Ester ((***S***,***R***)**-21**)

Triester (***S***,***R***)**-21** was obtained in a similar manner as (***S***,***S***)**-21** starting from triester (***S***,***R***)**-22**.

Recrystallization from TBME gave (***S***,***R***)**-21** as a white solid (yield 88.3%). This material can be additionally purified by chromatography (SiO_2_, CH_2_Cl_2_-MeOH, 99:1 → 93:7, *v*/*v*).

TLC: *R*_F_ = 0.70 (CH_2_Cl_2_/MeOH 10:1.4);

${\left[\text{α}\right]}_{\text{D}}^{20}$ = −9.56 (c = 1.0 MeOH);

^1^H-NMR δ: 10.60 (1H, d, *J* = 2.0 Hz, H9); 10.14 (1H,s, H1); 8.35 (1H, d, *J* = 7.8 Hz, H23); 8.32 (1H, d, *J* = 7.9 Hz, H17); 7.79 and 7.28 (2 × 2H, AA′BB′, H13 and H14); 6.3 (1H, m, H8); 6 (2H, s, H2); 4.48 (1H, m, H18); 4.24 (1H, ddd, *J* = 5.3 Hz, *J* = 7.8Hz, *J* = 9.3 Hz, H24); 4.10–4.00 (6H, 3 × m, ov, 3 × -CH_2_CH_3_); 2.97 (2H, m, H11); 2.85 (2H, m, H10); 2.38 (2H, m, ov, H20); 2.34 (2H, m, ov, H26); 2.02 (1H, m, ov, H19); 2 (1H, m, ov, H25); 1.96 (1H, m, ov, H19); 1.84 (1H, m, H25); 1.18–1.14 (9H, 3 × t, ov, *J* = 7.1 Hz, 3 × -CH_2_CH_3_);

^13^C-NMR δ: 172.4 (C21); 172.2 (C27); 171.7 (C22); 171.5 (C28); 166.4 (C16); 159.4 (C6); 152.2 (C2); 151.4 (C4); 146.2 (C12); 131.4 (C15); 128.1 (C13); 127.5 (C14); 117.7 (C7); 113.5 (C8); 98.8 (C5); 60.6 (-CH_2_CH_3_); 59.9 (2C, -CH_2_CH_3_); 52.7 (C18); 51.2 (C24); 36.2 (C11); 30.4 (C20); 29.8 (C26); 28.0 (C10); 27.0 (C19); 25.9 (C25); 14.0 (2C, -CH_2_CH_3_); 13.9 (‑CH_2_CH_3_);

^15^N-NMR δ: −208.0 (N3), −236.3 (N1); −241.4 (N9); −264.8 (N23); −266.0 (N17); −311.1 (C2-NH_2_);

HRMS calcd for [M + Na]^+^ C_31_H_40_N_6_O_9_Na *m*/*z* = 663.2754, found *m*/*z* = 663.2734.

#### 3.6.5. (2*S*)-2-[[(2*S*)-2-[[4-[2-Amino-4-oxo-4,7-dihydro-1*H*-pyrrolo[2,3-*d*]pyrimidin-5-yl)ethyl]benzoyl]amino]-4-carboxybutanoyl]amino]pentanedioic Acid ((***S***,***S***)**-9**)

Triester (***S***,***S***)**-21** (100 mg, 0.156 mmol) was treated with 1 M NaOH_aq_ (6 mL), the mixture was stirred at *RT*. After 30 min the reaction was completed (TLC control) and the mixture was acidified with 1 M HCl_aq_ to pH ≈ 3. The formed precipitate was filtered off, washed with water and dried (vacuum drier, 38 °C, overnight) to obtain (***S***,***S***)**-9** (58 mg, 66.8%).

TLC: *R*_F_ = 0.37 (2×CHCl_3_-MeOH 1:2); R_F_ = 0.50 (CHCl_3_-MeOH-H_2_O-25% NH_3aq_ 40:40:10:2)

${\left[\text{α}\right]}_{\text{D}}^{20}$ = +18.9 (c = 1, DMSO);

Mp. 171.5 °C;

^1^H-NMR δ: 10.60 (1H, s, H9); 10.17 (1H, s, H1); 8.34 (1H, d, *J* = 7.8 Hz, H17); 8.21 (1H, d, *J* = 7.8 Hz, H23); 7.78 (2H, BB′, H14); 7.28 (2H, AA′, H13); 6.31 (1H, m, H8); 6.01 (2H, s, C2-NH2); 4.46 (1H, m, H18); 4.22 (1H, m, H24); 2.97 (2H, m, H11); 2.85 (2H, m, H10); 2.34 (2H, m, H20); 2.30 (2H, m, H26); 2.03 (1H, ov, m, H19); 1.99 (1H, ov, m, H25); 1.92 (1H, m, H19); 1.81 (1H, m, H25);

^13^C-NMR δ: 174.3 (C21); 173.9 (C27); 173.3 (C28); 171.7 (C22); 166.6 (C16); 159.5 (C6); 152.3 (C2); 151.4 (C4); 146.2 (C12); 131.5 (C15); 128.2 (C13); 127.5 (C14); 117.5 (C7); 113.6 (C8); 98.8 (C5); 52.8 (C18); 51.3 (C24); 36.2 (C11); 30.5 (C20); 30.1 (C26); 28.1 (C10); 27.1 (C19); 27.1 (C19); 26.3 (C25); 26.3 (C25);

^15^N-NMR δ: −208 (N3); −236.3 (N1); −241.4 (N9); −263.2 (N23); −265.5 (N17); −311.1 (C2-NH2);

HRMS calcd for C_25_H_29_N_6_O_9_ *m*/*z* = 557.1996, found *m*/*z* = 557.1989.

FT-IR: [cm^−1^] 3315 (N-H_ν_, O-H_ν_); 2927 (C-H_ν_); 1702 (C=O_ν_); 1636 (C=O_ν_, C=N_ν_); 1534 (N-H_δ_, C=N_ν_); 1504 (C=C_ν_); 1405 (C-H_δ_); 1221 (C-O_ν_, C-N_ν_); 669 (C-H_γ_, N-H_γ_).

#### 3.6.6. (2*S*)-2-[[(2*R*)-2-[[4-[2-Amino-4-oxo-4,7-dihydro-1*H*-pyrrolo[2,3-*d*]pyrimidin-5-yl)ethyl]benzoyl]amino]-4-carboxybutanoyl]amino]pentanedioic Acid ((***S***,***R***)**-9**)

Triester (***S***,***R***)**-21** was hydrolyzed in the same manner as (***S***,***S***)**-21** ( yield of (***S***,***R***)**-21**, 47.8%).

TLC: *R*_F_ = 0.37 (2 × CHCl_3_-MeOH 1:2); R_F_ = 0.50 (CHCl_3_-MeOH-H_2_O-25% NH_3aq_ 40:40:10:2)

${\left[\text{α}\right]}_{\text{D}}^{20}$ = −23.46 (c = 0.8, DMSO);

Mp. 156.7 °C;

^1^H-NMR δ: 10.60 (s, C9); 10.15 (1H, s, C1); 8.30 (1H, d, *J* = 8.1 Hz, C17); 8.20 (1H, d, *J* = 7.9 Hz, C24); 7.79 (2H, BB′, C14); 7.28 (2H, AA′, C13); 6.32 (m, C8); 6.01 (2H, s, C2-NH2); 4.50 (1H, m, C18); 4.23 (1H, m, C25); 2.98 (2H, m, C11); 2.86 (2H, m, C10); 2.32 (2H, m, C21); 2.25 (2H, m, C28); 2.01 (1H, m, ov, C20); 1.99 (1H, m, ov, C27); 1.94 (1H, m, ov, C19); 1.79 (1H, m, C26);

^13^C-NMR δ: 174.1 (C22); 173.7 (C29); 173.1 (C30); 171.6 (C23); 166.4 (C16); 159.3 (C6); 152.2 (C2); 151.3 (C4); 146.1 (C12); 131.5 (C15); 128.1 (C13); 127.5 (C14); 117.7 (C7); 113.4 (C8); 98.7 (C5); 52.8 (C18); 51.1 (C25); 36.1 (C11); 30.5 (C21); 29.9 (C28); 28 (C10); 27.2 (C19); 27.2 (C20); 26.4 (C26); 26.4 (C27);

HRMS calcd for C_25_H_29_N_6_O_9_ *m*/*z* = 557.1996, found *m*/*z* = 557.1989.

### 3.7. Synthesis of (2S,2′S)-2,2′-[[2,2′-Diamino-4,4′,6-trioxo-1,4,4′,6,7,7′-hexahydro-1′H,5H-5,6′-bipyrrolo[2,3-*d*]pyrimidine-5,5′-diyl]bis(ethylenebenzene-4,1-diylcarbonylimino)]dipentanedioic Acid (Impurity **10**)

Pemetrexed disodium heptahydrate (**1a**, 2.0 g) was dissolved in 0.1 M NaOH_aq_ (400 mL) and heated under reflux for 3 days (TLC control). Then the mixture was cooled and evaporated under reduced pressure to get crude diastereoisomeric mixture **10** as brown oil.

The obtained mixture was purified by chromatography (SiO_2_, EtOH-MeOH-AcOEt-4%NH_3 aq_, 40:30:10:12, *v*/*v*).The respective fractions were collected and concentrated. The residue was dissolved in water (10 mL) and the pH was adjusted to 2–3 with 1 M HCl_aq_. The suspension was filtered, then the solid was washed with H_2_O (2 × 2 mL) and dried at 40 °C to obtain **10** (407 mg, 10%).

TLC: *R*_F_ = 0.59 (EtOH/MeOH/AcOEt/4%NH_3 aq_ 40:30:10:12 *v*/*v*)

HPLC purity 84.3%

Mp. 230 °C

HRMS: calcd for C_40_H_39_N_10_O_13_ *m*/*z* = 867.2698, found *m*/*z* = 867.2709

^1^H-NMR δ: 12.39 (3H, ov, total for 21, 21′, 22 and 22′ –CO_2_H), 10.87 (1H, s, N9′-H), 10.74 (1H, s, N9-H), 10.60 (1H, bs, probably N1′-H), 10.09 (1H, s, N1-H), 8.52 (2H, 2 × d, ov, N17-H and N17′-H), 7.80 (4H, m, H14 and H14′), 7.30 (2H, d, *J* = 8.0 Hz, H13′), 7.26 (2H, d, *J* = 8.0 Hz, H13), 6.90 (2H, bs, probably NH_2_ at C2′), 6.02 (2H, NH_2_ at C2), 4.41 (2H, m, H18 and H18′), 2.72–2.54 (6H, H11, H10 and H10′), 2.45 (2H, H11′), 2.40–2.32 (4H, H20 and H20′), 2.10 and 1.97 (4H, H19 and H19′);

^13^C-NMR δ: 179.59 (C8′), 173.93, 173.90 (CO, C21 and C21′), 173.51 and 173.48 (CO, C22, C22′), 166.59 and 166.41 (CO, C16 and C16′), 164.03 (probably C4′), 159.08 (C6), 157.76 (probably C6′), 157.63 (unknown, C4 or C2′), 152.10 (unknown, C4 or C2′), 150.29 (probably C2), 146.52 (C12), 145.00 (C12′), 131.49 and 131.39 (C15 and C15′), 128.08 (C13′), 127.97 (C13), 127.54 and 127.42 (C14 and C14′), 114.22 (C7), 99.51 (C5), 92.71 (C5′), 51.91 (C18 and C18′), 51.72 (C7′), 37.76 (C11), 34.18 (C10′), 30.44 (C20, C20′), 29.75 (C11′), 28.08 (C10), 25.98 (C19 and C19′);

^15^N-NMR δ: −311.0 (NH_2_ at C2), −265.9 (N17 and N17′), −238.9 (N9), −236.2 (N1), −233.7 (N9′), all remaining ^15^N-NMR signals not recorded in the ^1^H-^15^N *g*-HMBC experiment.

## 4. Conclusions

Herein we have developed and described the synthesis of the process-related impurities of pemetrexed disodium, the active ingredient of an anticancer therapeutic. The process related impurities **6**, **7** and **10** we synthesized by modified methods. For the impurities **8** and **9** we developed new synthetic methods. We found that during synthesis the impurity **9** a mixture of diastereoisomers can be formed. To avoid this process, we have developed an effective method of the synthesis of **9** where racemization does not occur. Two diastereoisomeric impurities **9** were obtained: ***S****,**S***-**9** and, reported for the first time, ***S****,**R*****-9**.

The structure elucidation of all obtained impurities was discussed on the basis of two-dimensional NMR experiments and MS data and their physicochemical characterization was presented. We have developed HPLC methods for the determination of chemical and enantiomeric purity of pemetrexed disodium and its impurities.

The determination of the impurity profile and elucidation of the structures of the main contaminants are of great importance when it comes to complying with the regulatory norms as well as assessing the quality of pemetrexed disodium as an API.

## Supplementary Material

The IR characteristic of impurities **6**, **7**, **8** and **9**, as well as experimental procedures and analytical data for the sodium salts of **7** and **8** and the mixture of diastereoisomers of **9** are collected in the supporting materials which can be accessed at: http://www.mdpi.com/1420-3049/20/06/10004/s1.
