# Supplementary material for: Synthesis and Physicochemical Characterization of the Impurities of Pemetrexed Disodium, an Anticancer Drug
Source: Molecules. 2015 May 29;20(6):10004–31. doi: 10.3390/molecules200610004 (PMC6272243; doi:10.3390/molecules200610004)
Supplement: Supplementary file 1 [file molecules-20-10004-s001.pdf]

Supplementary

Synthesis and Physicochemical Characterization of the Impurities of Pemetrexed Disodium, an Anticancer Drug

Olga Michalak, Mariusz M. Gruza, Anna Witkowska, Iwona Bujak and Piotr Cmoch

1. Chiral HPLC for (2*R*)-2-[[4-[2-(2-Amino-4-oxo-4,7-dihydro-1*H*-pyrrolo[2,3-d]pyrimidin-5-yl)ethyl]benzoyl]amino]-pentanedioic Acid (*R*)-1

Chromatographic analysis was performed using Waters® Alliance HPLC system (Waters Co., Milford, MA, USA) consisting of Waters® e2695 Separation Module and Waters® 2998 Photodiode Array Detector. Separation of the analyte from potential impurities was achieved using a Gemini C18 column (250 mm × 4.6 mm, 5 µm, Phenomenex, Torrance, CA, USA) placed in a thermostated column heater at 40 °C. The mobile phase consisting of 8 g/L β-cyclodextrin buffer at pH 6.0:acetonitrile (95:5 *v*/*v*) was used in isocratic mode at the flow rate of 1 mL/min. The samples were prepared at a concentration of about 0.24 mg/mL and they were diluted in water. The injection volume was 50 µL. The UV detection at 230 nm was used.

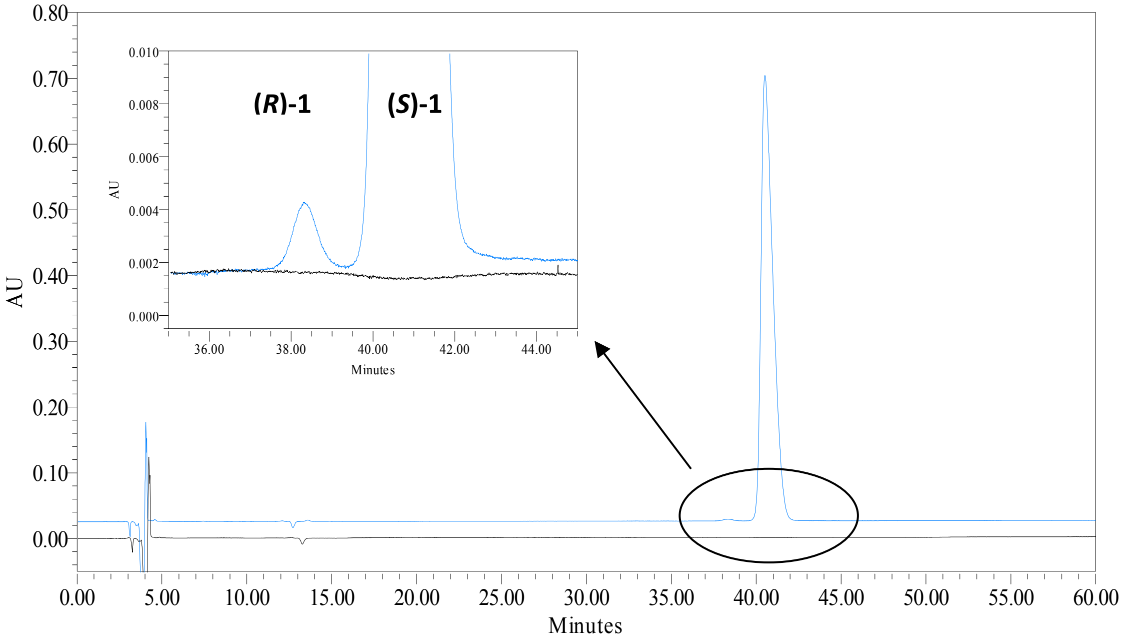

Figure S1. Chromatogram for the test for enantiomeric purity of Pemetrexed Disodium. **(*S*)-1**—Pemetrexed Disodium; **(*R*)-1**—enantiomeric impurity.

2. Synthesis of Sodium Salt Forms of Impurities 7 and 8

2.1. General Information

The starting 4-[2-(2-amino-4-oxo-4,7-dihydro-1H-pyrrolo[2,3-d]pyrimidin-5-yl)ethyl]benzoic acid (**2**) was obtained from Neorganic, Warsaw, Poland. γ-Ethyl L- and D-glutamates were prepared according to the literature procedure [1]. Other materials, solvents and reagents were of commercial

origin and used without additional operations. All reactions were carried out in ambient temperature, if not stated otherwise. Coupling reactions were performed in anhydrous solvents.

The purity of the examined compounds was determined using HPLC/UHPLC methods with the chromatography system UltiMate™ 3000RS UHPLC (Dionex Corporation, Sunnyvale, CA, USA) equipped with an autosampler and a DAD 3000RS detector.

Method A: Gemini C18 column (150 mm × 4.6 mm, 3µm, Phenomenex) was placed in a thermostated column heater at 25 °C. The mobile phases consisting of A (4 g/L dipotassium hydrogen phosphate; pH 5.2) and B (acetonitrile) were used with the gradient mode at the flow rate of 0.9 mL/min. The samples were prepared at a concentration of about 0.5 mg/mL and were diluted in 0.4 g/L of dipotassium hydrogen phosphate. The injection volume was 10 µL. The UV detection at 230 nm was used.

The TLC separations were performed on the TLC silica gel 60 F254 on alumina sheets (Merck and/or Sigma-Aldrich). The visualization was performed by UV light (254 and/or 365 nm).

The specific rotation $[\alpha]_D$ was calculated from an optical rotation measurement performed on the Perkin Elmer 341 Polarimeter (PerkinElmer, Waltham, MA, USA) at the wavelength of 589 nm (D line of a sodium lamp), at 20 °C.

The melting points were determined by differential scanning calorimetry (DSC) carried out by means of the DSC822 with an IntraCooler (Mettler Toledo GmbH, Schwerzenbach, Switzerland).

The ^{1}H and ^{13}C-NMR spectra were recorded in the DMSO-d_6 or D_2O solutions with the Varian-NMR-vnmrs600 spectrometer (Varian Inc. Palo Alto, CA, USA) at 298 K temperature, equipped with a 600 MHz PFG Auto XID (^{1}H/^{15}N-^{31}P 5 mm) indirect probehead. To identify the structures of pemetrexed impurities correctly, careful analysis of the results of 1D and 2D NMR experiments was employed. The 1D and 2D measurements covered: ^{1}H selective NOESY, 2D: COSY, the ^{1}H-^{13}C gradient selected HSQC and HMBC optimized for ^{1}J(C-H) = 150 Hz and ^{n}J(C-H) = 8 Hz, respectively. The ^{15}N-NMR chemical shifts were obtained on the basis of the 2D ^{1}H-^{15}N gradient selected HSQC and HMBC experiments, optimized for ^{1}J(N-H) = 90 Hz and ^{n}J(N-H) = 6 Hz, respectively. Standard experimental conditions and standard Varian programs (ChemPack 4.1) were used. The ^{1}H and ^{13}C-NMR chemical shifts are given relative to the TMS signal at 0.0 ppm, whereas neat nitromethane at 0.0 ppm was used as a standard for the ^{15}N-NMR chemical shifts. The concentration of the solutions used for the measurements was about 20–30 mg of the compounds in 0.6 cm^3 of deuterated solvent. Used abbreviations: s—singlet, d—doublet, t–triplet, m—multiplet, ov—overlapped signals. The integrals are not presented due to the signals overlapping in most cases. Some ^{1}H-NMR chemical shifts for all the compounds studied are given as the averaged value of the center of multiplets read from the ^{1}H-^{13}C g-HSQC experiments.

The mass spectra were recorded on the MaldiSYNAPT G2-S HDMS (Waters Co., Milford, MA, USA) Spectrometer via electrospray ionization (ESI-MS).

2.2. (2S)-2-[[4-[2-(2-(dimethylamino)methyleneamino-4-oxo-4,7-dihydro-1H-pyrrolo[2,3-d]pyrimidin-5-yl)ethyl]benzoyl]amino]pentanedioic acid disodium salt (7a)

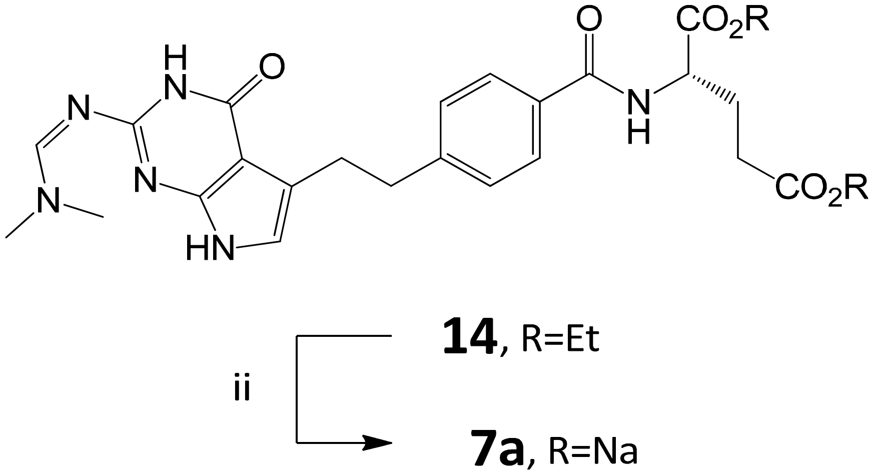

Scheme S1. Synthesis of salt **7a**.

Compound **14** (0.93 g, 1.12 mmol) was treated with 1M $NaOH_{aq}$ (10 mL), the mixture was cooled to 0–5 °C and stirred for 15 min. EtOH (80 mL) was added to the solution and stirred for 1 h at 0–5 °C. The pH was adjusted to 7–8 with 1 M HCl_{aq} (15 mL). EtOH (220 mL) was added and stirred for 30 min. The suspension was filtered and the solid washed with EtOH (10 mL) and dried at 40 °C, to obtain **7a**. The solid was suspended in MeOH (22 mL) at 0–5 °C and stirred for 60 min. The MeOH purification was repeated and the product was dried at 35 °C under vacuo to obtain **7a** (0.67 g, 80.0%, HPLC purity 97.1%).

TLC: R_F = 0.49 ($CHCl_3$-MeOH, 1:2);

$[\alpha]_D^{20}$ = +7.08 (c = 1, DMSO);

^{1}H-NMR (D_2O) : 8.03 (1H, s, -N=CH-N(CH_3)$_2$), 7.68 (2H, m, H14), 7.18 (2H, m, H13), 6.38 (1H, s, H8), 4.35 (1H, m, H18), 3.02 (3H, s, one of the CH_3 groups of -N=CH-N(CH_3)$_2$), 2.89 (3H, s, one of the CH_3 groups of -N=CH-N(CH_3)$_2$), 2.75 (4H, both H10 and H11 protons), 2.32 (2H, m, both H20 protons), 2.18 (1H, m, one of the H19 protons), 2.05 (1H, m, one of the H19 protons);

^{13}C-NMR (D_2O) : 184.95 (CO, C21), 181.60 (CO, C22), 172.38 (CO, C16), 164.25 (probably C6), 160.23 (-N=CH-N(CH_3)$_2$), 157.59 (C2), 152.74 (probably C4), 149.4 (C12), 133.46 (C15), 131.19 (C13), 129.78 (C14), 120.84 (C7), 119.34 (C8), 103.96 (C5), 58.65 (C18), 43.74 (one of the CH_3 groups of -N=CH-N(CH_3)$_2$), 38.11 (C11), 37.32 (one of the CH_3 groups of -N=CH-N(CH_3)$_2$), 36.96 (C20), 31.26 (C19), 29.53 (C10);

^{15}N-NMR (D_2O): −273.2 (-N=CH-N(CH_3)$_2$), −256.6 (N17), −242.2 (N9), −188.0 (-N=CH-N(CH_3)$_2$), N1 and N3 not recorded in ^{1}H-^{15}N *g*-HMBC experiments;

HRMS: calcd for $C_{23}H_{25}N_6O_6$ *m/z* = 481.1836, found *m/z* = 481.1852.

2.3. (2S)-2-[[(4S)-4-[[4-[2-amino-4-oxo-4,7-dihydro-1H-pyrrolo[2,3-d]pyrimidin-5-yl)ethyl]benzoyl]amino]-4-carboxybutanoyl]amino]pentanedioic acid trisodium salt (8a)

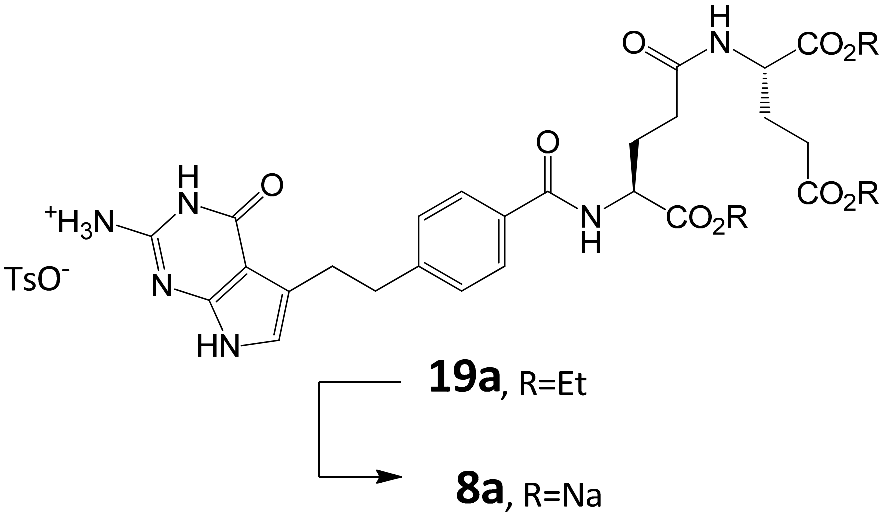

Scheme S2. Synthesis of salt **8a**.

Compound **19a** (0.97 g, 1.17 mmol) was treated with 1 M $NaOH_{aq}$ (10 mL), the mixture was stirred at *RT*. After 1 h the reaction mixture was adjusted to pH 8.0 with 1 M HCl_{aq} and lyophilized. The residue was dissolved in water (4 mL) and was heated to 50–60 °C. EtOH (20 mL) was added to the solution and cooled to *RT*. The oil layer was separated by decantation and was treated with EtOH (25 mL). After 2 h stirring at *RT*, the solid was collected by filtration, washed with EtOH (1 × 10 mL) and dried in vacuo at 40 °C for 24 h to obtain trisodium salt **8a** (0.59 g, 81%), HPLC purity 89.7%.

TLC: R_F = 0.39 (developed twice in $CHCl_3$-MeOH 1:2); R_F = 0.37 ($CHCl_3$-MeOH-H_2O-25% NH_{3aq} 40:40:10:2);

^{1}H-NMR (D_2O): 7.67 (2H, m, H14), 7.20 (2H, m, H13), 6.34 (1H, s, H8), 4.38 (1H, m, H18), 4.11 (1H, m, H24), 2.83 (5H, m, H11, H12 and one of H19 protons), 2.45 (2H, m, both H20 protons), 2.23 (2H, m, both H26 protons), 2.11 (1H, m, and one of H19 protons), 2.06 (1H, m, and one of H25 protons), 1.89 (1H, m, and one of H25 protons);

^{13}C-NMR (D_2O): 184.92 (CO, C27), 181.70 (CO, C28), 181.31 (CO, C22), 177.82 (CO, C21), 172.77 (CO, C16), 163.92 (C6), 154.79 (probably C2), 153.52 (probably C4), 149.52 (C12), 133.36 (C15), 131.34 (C13), 129.76 (C14), 120.81 (C7), 118.34 (C8), 101.52 (C5), 58.15 (C18), 58.09 (C24), 38.25 (C11), 36.86 (C26), 35.20 (C20), 31.19 (C25), 30.50 (C19), 29.61 (C10);

HRMS: calcd for $C_{25}H_{28}N_6O_9Na$ *m/z*= 579.1815, found *m/z*=579.1805.

3. Synthesis of Impurity 9 (by Monoester)

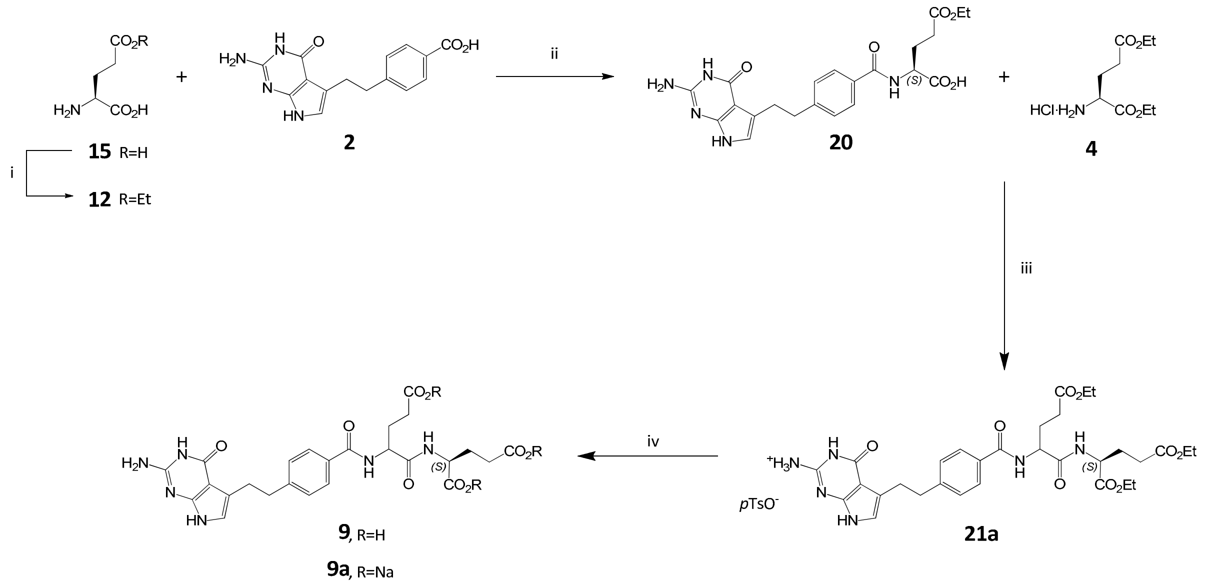

Scheme S3. Synthesis of impurity **9**. Conditions: (i) HCl, EtOH; (ii) CDMT, NMM, DMF; (iii) (a) CDMT, NMM, DMF; (b) *p*-TsOH, EtOH; (iv) (a) 1 M $NaOH_{aq}$, (b) 1 M HCl_{aq}.

3.1. L-Glutamic Acid γ-Ethyl Ester (12)

L-Glutamic acid **15** (50 g, 340 mmol) was added to ethanol containing hydrogen chloride (HCl in EtOH, 1.42 mol/L, 350 mL, 497 mmol), and the mixture kept for 2 days. Pyridine (53.25 mL, 661 mmol) was added. The ester was collected and washed with EtOH. Recrystallization from EtOH/H_2O (95:5) and dried in vacuo gave **12** as white solid (16.64 g, 28%).

TLC: $R_F = 0.75$ ($CHCl_3$-MeOH-NH_3, 2:2:1)

Mp. 181°C, (lit. [1] 195–198 °C);

$[\alpha]_D^{20}$ = +27.1 (c = 1, 1 M HCl); (lit. [1] $[\alpha]_D^{20}$ = +32.1 (c = 2, 1 N HCl); $[\alpha]_D^{20}$ = +12.8 (c=1, H_2O),)

^{1}H-NMR (DMSO) : 8.50 (3H, broad s, N$\underline{H}_2$ and COO$\underline{H}$), 4.07 (2H, q, J = 7.1 Hz, -OC$\underline{H}_2$-CH_3), 3.71 (1H, t, J = 6.6 Hz, -C$\underline{H}$-NH_2), 2.55 (1H, ddd, J = 6.1, 10.0, 16.2 Hz, one of C$\underline{H}_2$β), 2.44 (1H, ddd, J = 6.1, 10.0, 16.2 Hz, one of C$\underline{H}_2$β), 2.02 (2H, m, both CH_2α), 1.19 (3H, t, J = 7.1 Hz, -OCH_2-C$\underline{H}_3$);

^{13}C-NMR (DMSO): 171.91 (COγ), 170.63 (COα), 60.00 (-O$\underline{C}H_2$-CH_3), 51.77 (-$\underline{C}$H-NH_2), 29.50 (CH_2β), 25.50 (CH_2α), 14.07 (-OCH_2-$\underline{C}H_3$);

^{15}N-NMR (DMSO): −338.7.

HRMS calcd for $C_7H_{14}NO_4$ *m/z* = 176.0923, found *m/z* = 176.0926

3.2. (2S)-2-[[4-[2-(2-Amino-4-oxo-4,7-dihydro-1H-pyrrolo[2,3-d]pyrimidin-5-yl)ethyl]benzoyl]amino]-pentanedioic acid 5-ethyl ester (20)

To a suspension of acid **2** (1.5 g, 5.03 mmol) in DMF (15 mL) was added *N*-methylmorpholine (NMM, 1 mL, 9.1 mmol) followed by 2-chloro-4.6-dimethoxy-1,3,5-triazine (CDMT, 0.98 g, 5.55 mmol), and the resulting solution was stirred at *RT* for 3 h. Then to the solution L-glutamic acid γ-ethyl ester (**12**) (0.86 g, 4.9 mmol) was added and the resulting mixture was stirred for another 3 h. The reaction

mixture was poured into water (20 mL) and extracted with dichloromethane (3 × 20 mL). The organic phases were collected, dried over anhydrous magnesium sulfate and concentrated to give of product **20** as a yellow solid (1.38 g, 60%), used without purification in a subsequent step.

An analytical sample was prepared separately by chromatography on silica gel.

TLC R_F = 0.46 ($CHCl_3$/MeOH, 4:6)

^{1}H-NMR (DMSO) : 10.86 (1H, broad s, N1-H), 10.63 (1H, s, N9-H), 8.16 (1H, d, *J* = 6.8 Hz, N17-H), 7.78 (2H, d, *J* = 7.8 Hz, H14), 7.30 (2H, d, *J* = 7.8 Hz, H13), 6.38 (2H, s, NH_2 at C2), 6.32 (1H, s, H8), 4.31 (1H, m, H18), 4.02 (2H, q, *J* = 7.1 Hz, O-CH_2-CH_3), 2.99 (2H, m, H11), 2.87 (2H, m, H10), 2.40 and 2.35 (2H, 2 × m, both H20), 2.15 (1H, m, one of H19), 1.99 (1H, m, one of H19), 1.15 (3H, t, *J* = 7.1 Hz, O-CH_2-CH_3);

^{13}C-NMR (DMSO) : 174.34 (CO, C22); 172.76 (CO, C21), 165.85 (CO, C16), 159.67 (probably C6), 152.60 (probably C2), 151.53 (probably C4), 146.00 (C12), 131.86 (C15), 128.22 (C13), 127.16 (C14), 117.65 (C7), 113.35 (C8), 98.70 (C5), 59.75 (O-CH_2-CH_3), 53.00 (C18), 36.24 (C11), 30.47 (C20), 28.15 (C10), 27.15 (C19), 14.07 (O-CH_2-CH_3);

^{15}N-NMR (DMSO): −310.2 (NH_2 at C2), −263.5 (N17), −241.7 (N9), −235.7 (probably N1-H), −208.5 (probably N3).

HRMS calcd for $C_{22}H_{24}N_5O_6$ *m/z* = 454.1742, found *m/z* = 454.1732.

3.3. (2S)-2-[(2S)-2-[4-(2-(2-amino-4-oxo-4,7-dihydro-1H-pyrrolo[2,3-d]pyrimidin-5-ylo)-ethyl]benzoyl]amino]-4-(ethoxycarbonyl)butanoyl]amino]pentanedioic acid diethyl ester (21)

From diester **4** and monoester **20**.

To a suspension of **20** (1.83 g, 4.0 mmol) in DMF (20 mL) was added NMM (1.23 mL, 11.2 mmol), followed by CDMT (0.85 g, 4.82 mmol), and the resulting solution was stirred at *RT* for 3 h. At this point, was added to this solution L-glutamic acid diethyl ester hydrochloride (**4**, 1.06 g, 4.4 mmol), and the resulting mixture was stirred for 24 h. To the reaction mixture were added water (20 mL) and methylene chloride (20 mL), and the mixture was stirred for 15 min. The organic layer was separated and the aqueous phase extracted with dichloromethane (2 × 20 mL). The organic layers were collected, washed with 1 M $NaHCO_3$ (1 × 25 mL), and concentrated under reduced pressure to afford an oil.

To the oil was added EtOH (30 mL) followed by a solution of p-toluenesulfonic acid monohydrate in EtOH (1.9 g in 30 mL) and the resulting suspension was heated under reflux for 2 h. The mixture was cooled to *RT*, the crystals of **21a** were filtered and washed with EtOH (2 × 10 mL). The wet cake was reslurried in EtOH (30 mL), refluxed for 1.5 h and cooled to *RT*. The crystals were filtered, washed with EtOH (2 × 5 mL) and dried in vacuo at 40°C for 24 h to provide **21a** (1.43 g, 43%).

TLC R_F = 0.41 ($CHCl_3$/MeOH 8:2)

p-TSA salt

Mp. 227 °C

^{1}H-NMR (DMSO) : 10.70 (1H, s, N9-H), 10.33, 8.38, 8.36 and 8.33 (2H, three overlapped d and d, N23-H and N17-H), 7.80 (2H, two overlapped d, H14), 7.52 (2H, d, *J* = 8.0 Hz, protons of *p*-TSA), 7.29 (2H, two overlapped d, H13), 7.14 (2H, d, *J* = 8.0 Hz, protons of *p*-TSA), 6.34 (1H, s, H8), 6.25 (2H, broad s, NH_2 at C2), 4.47 (1H, m, H18), 4.26 (1H, m, H24), 4.10–4.00 (6H, m, CH_2 protons of all -O-CH_2-CH_3 groups), 2.98 (2H, m, H11), 2.86 (2H, m, H10), 2.45–2.37 (3H, m, both

protons H20 and one of the H26 protons), 2.35 (1H, m, one of the H26 protons), 2.10–1.91 (3H, m, one of the H25 protons and both H19 protons), 1.86 (1H, m, one of the H25 protons), 1.20–1.12 (9H, m, CH_3 protons of all -O-CH_2-C$\underline{H}_3$ groups);

^{13}C-NMR (DMSO) : 172.41 and 172.36 (CO, C21), 172.17 and 172.14 (CO, C27), 171.65 and 171.61 (CO, C22), 171.47 and 171.42 (CO, C28), 166.38 and 166.36 (CO, C16), 159.00 (probably C6), 151.97 (probably C2 and C4), 146.07 and 146.04 (C12), 145.21 and 137.94 (C_{IV} of Ph ring of *p*-TSA), 131.39 (C15), 128.14 (CH of Ph ring of *p*-TSA), 128.07 (C13), 127.47 (C14), 125.53 (CH of Ph ring of p-TSA), 117.82 (C7), 113.67 (C8), 98.76 (C5), 60.60, 60.58, 59.91 and 59.87 (all CH_2 carbons of -O-CH_2CH_3 groups), 52.65 and 52.50 (C18), 51.23 and 51.20 (C24), 36.08 (C11), 30.32 C20), 29.76 and 29.72 (C26), 26.96 and 26.87 (C19), 25.90 and 25.84 (C25), 20.78 (CH3 of p-TSA), 14.06, 14.04, 13.93 and 13.92 (all CH_3 carbons of -O-CH_2CH_3 groups);

^{15}N-NMR (DMSO): not recorded.

HRMS calcd for $C_{31}H_{40}N_6O_9Na$ *m/z* = 663.2749, found *m/z* = 663.2754.

3.4. (2S)-2-[(2S)-2-[4-(2-(2-Amino-4-oxo-4,7-dihydro-1H-pyrrolo[2,3-d]pyrimidin-5-ylo)ethyl]benzoyl]amino]-4-carboxybutanoyl]amino]pentanedioic acid (9)

3.4.1. Trisodium salt **9a**

Compound **21a** (0.93 g, 1.12 mmol) was treated with 1 M $NaOH_{aq}$ (10 mL), the mixture was stirred at *RT*. After 1 h the reaction mixture was adjusted to pH 8.0 with 1 M HCl_{aq} and heated to 50–60 °C. Acetone (85 mL) was added to the solution, which then cooled to *RT*. The precipitated solid was collected by filtration, washed with acetone (2 × 10 mL) and dried to obtain **9a** as trisodium salt (0.69 g, 96.9%).

HPLC purity 80.1%

TLC R_F =0.37 (2×$CHCl_3$-MeOH 1:2); R_F = 0.50 ($CHCl_3$-MeOH-H_2O-25% NH_{3aq} 40:40:10:2).

Sodium salt form

Mp.129°C

^{1}H-NMR (600 MHz, D_2O): 7.69 (2H, m, H14), 7.20 (2H, m, H13), 6.34 (1H, s, H8), 4.56 (1H, m, H18), 4.23 (1H, m, H24), 2.82 (4H, m, H10 and H11), 2.43 (2H, m, H20), 2.28 (2H, m, H26), 2.24 and 2.14 (3H, m, both H19 protons and one of H25), 1.97 (one of H25 protons);

^{13}C-NMR (150 MHz, D_2O): 184.94 and 184.82 (CO, C27), 184.54 and 184.46 (CO, C21), 181.35 and 181.30 (CO, CC28), 176.20 (CO, C22), 173.38 and 173.30 (CO, C16), 163.91 (probably C6), 154.78 (probably C2), 153.50 (probably C4), 149.80 and 149.78 (C12), 132.95 and 132.92 (C15), 131.33 and 131.23 (C13), 129.93 (C14), 120.86 (C7), 118.27 (C8), 101.55 (C5), 58.11 and 58.01 (C24), 57.37 and 57.32 (C18), 38.29 (C11), 36.78 and 36.75 (C26), 36.67 and 36.65 (C20), 31.38 and 31.36 (C25), 30.94 and 30.57 (C19), 29.61 (C10).

HRMS calcd for $C_{25}H_{28}N_6O_9Na$ *m/z* = 579.1815, found *m/z* =579.1808.

3.4.2. Triacid Form **9**

Compound **21a** (4.20 g, mmol) was treated with 1 M $NaOH_{aq}$ (30 mL), and the mixture was stirred at *RT*. After 4 h the reaction mixture was adjusted to pH 8.0 with 1N HCl_{aq} and precipitate was formed.

EtOH (40 mL) was added to the solution, the precipitate was dissolved in few minutes, then a new precipitation was formed. After 15 min of stirring the precipitated solid was collected by filtration, washed with EtOH (2 × 7 mL) and dried to obtain crude **9** as a solid (2.26 g). The crude material was purified by chromatography (SiO_2, $CHCl_3$-MeOH-H_2O-25% NH_{3aq} 40:40:5:2) to obtain main fraction of **9** (1.5 g, HPLC 98.0%). The obtained material was then dissolved in water (20 mL) and the solution was acidified to pH=3 with 10% HCl_{aq} (*ca.* 4.5 mL), forming a precipitate. The mixture was stirred at RT for 30 min, then the precipitate was filtered off, washed with water (3 × 15 mL) and dried to give **9** (in triacid form, 1.14 g, 39.6% from **21a**).

HPLC purity 96.8%

Triacid form.

Mp.152°C

^{1}H-NMR (DMSO) : 10.61 (1H, s, N9-H), 10.18 (1H, s, N1-H), 8.35 and 8.32 (1H, 2 × d, *J* = 7.9 Hz, N17-H), 8.21 and 8.19 (1H, 2 × d, ov N23-H), 7.80 and 7.79 (2H, m, H14), 7.29 (2H, m, H13), 6.32 (1H, s, H8), 6.03 (2H, s, NH_2 at C2), 4.49 and 4.47 (1H, 2 × m, ov, H18), 4.23 (1H, m, H25), 2.98 (2H, m, H11), 2.86 (2H, m, H10), 2.33 (2H, m, H20 and one of the H26 protons), 2.25 (1H, t, *J* = 7.5 Hz, H26), 2.03 and 1.93 (3H, m, both H19 protons and one of the H25 protons), 1.80 (1H, m, one of the H26 protons);

^{13}C-NMR (DMSO): 174.17, 174.11 (CO, C21), 173.83, 173.77 (CO, C29), 173.20, 173.12 (CO, C30), 171.61, 171.56 (CO, C22), 166.44, 166.42 (CO, C16), 159.32 (CO, C6), 152.23 (probably C2), 151.34 (probably C4), 146.14, 146.12 (C12), 131.49 (C15), 128.14 (C13), 127.47 (C14), 117.69, 117.67 (C7), 113.50 (C8), 98.76 (C5), 52.82, 52.67 (C18), 51.24, 51.20 (C25), 36.17 (C11), 30.50, 30.48 (C20), 30.07, 30.00 (C27), 28.04 (C10), 27.19, 27.02 (C19), 26.46, 26.30 (C26).

^{15}N-NMR (DMSO): −311.1 (NH_2 at C2), −265.7 (N17), −263.7 (N24), −241.3 (N9), −236.3 (N1), −208.0 (N3).

HRMS calcd for $C_{25}H_{27}N_6O_9$ *m/z* = 555.1840, found *m/z* =555.1843

4. IR characteristic of Impurities

4.1. General Information

Water content determination was carried out by Karl Fischer volumetric titration using the Methrom 701 KF Titrino apparatus (Mettler Toledo GmbH, Schwerzenbach, Switzerland) and the Methrom 6.0338.100 electrode.

FT-IR spectra were recorded on the Nicolet iS10 (Thermo Scientific) spectrometer in the range of 4000–400 cm^{-1} and with the spectral resolution of 4 cm^{-1} in KBr pellets (~1.5 mg of substance/200 mg KBr).

Raman spectroscopy

FT Raman spectra were recorded on the Nicolet NXR 9650 instrument (Thermo Scientific), using 1064 nm excitation from Nd:YVO4 laser, in the range from 150 to 3700 cm^{-1} with the spectral resolution of 4 cm^{-1}.

4.2. Spectra

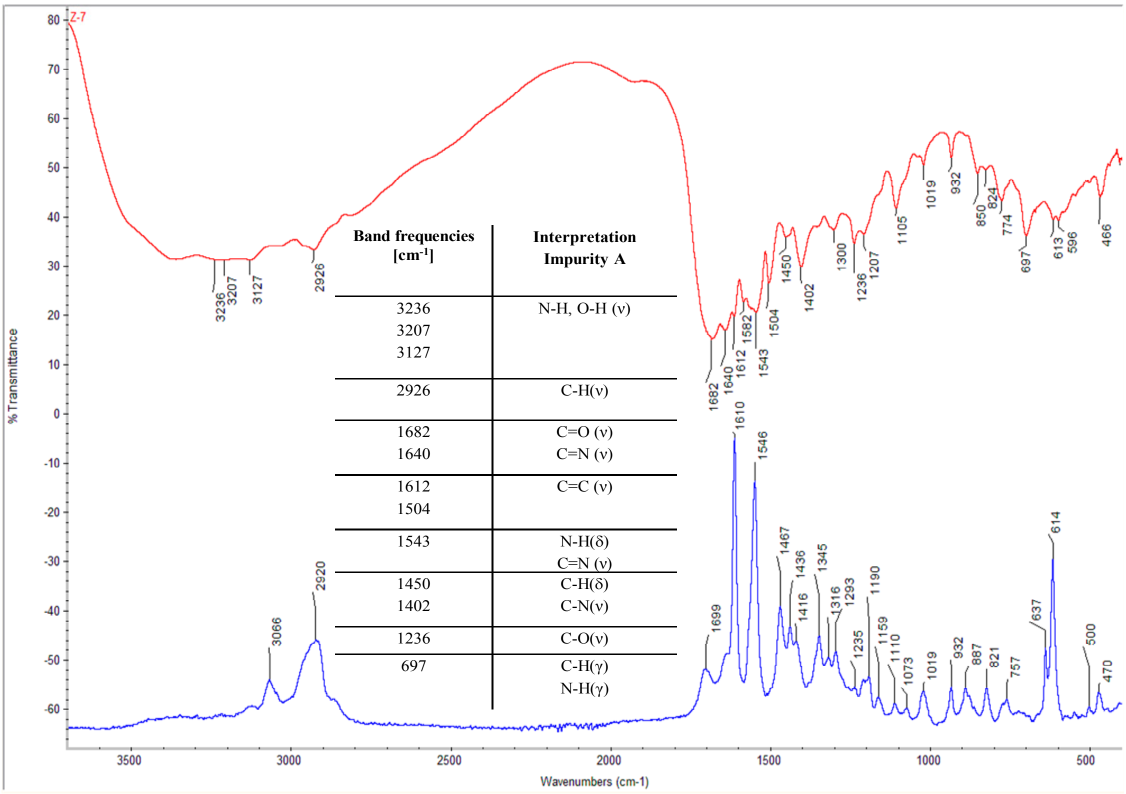

Band frequencies [cm^{-1}]	Interpretation Impurity A
3236<br>3207<br>3127	N-H, O-H (ν)
2926	C-H(ν)
1682<br>1640	C=O (ν)<br>C=N (ν)
1612<br>1504	C=C (ν)
1543	N-H(δ)<br>C=N (ν)
1450<br>1402	C-H(δ)<br>C-N(ν)
1236	C-O(ν)
697	C-H(γ)<br>N-H(γ)

Figure S6. Raman and IR spectra of **6** (impurity A).

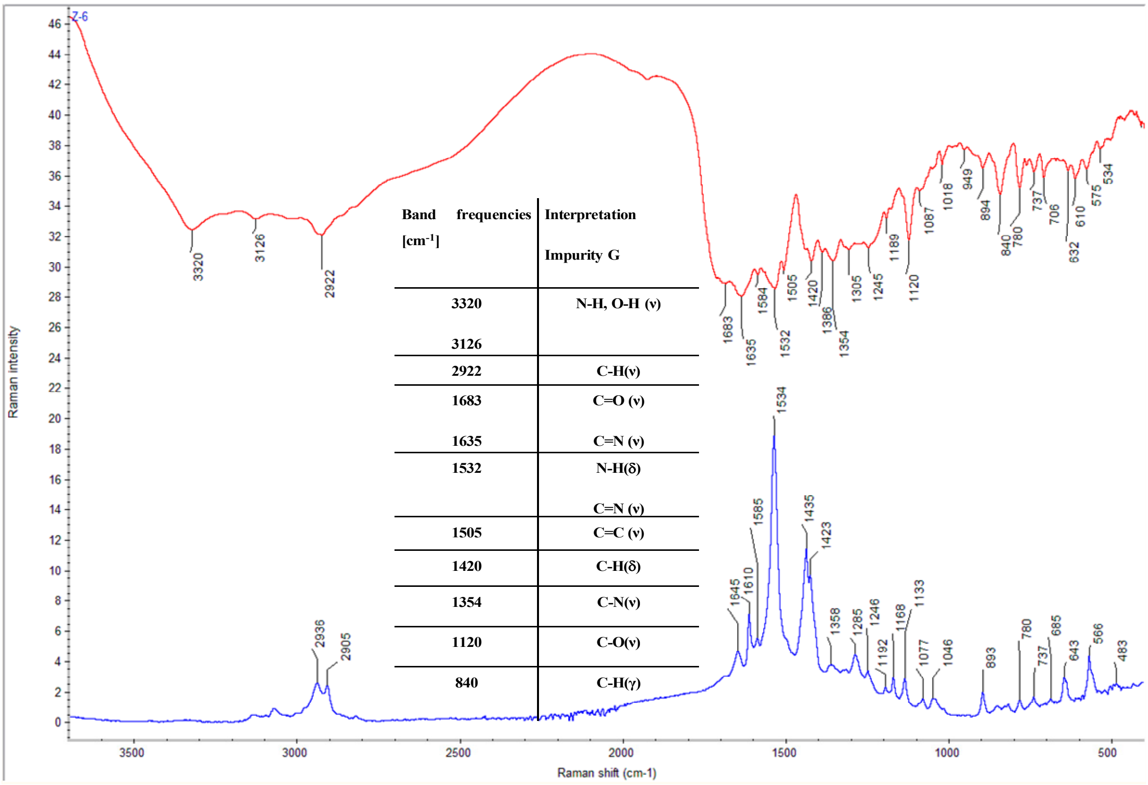

Band frequencies [cm^{-1}]	Interpretation Impurity G
**3320**<br>**3126**	**N-H, O-H (ν)**
**2922**	**C-H(ν)**
**1683**<br>**1635**	**C=O (ν)**<br>**C=N (ν)**
**1532**	**N-H(δ)**<br>**C=N (ν)**
**1505**	**C=C (ν)**
**1420**	**C-H(δ)**
**1354**	**C-N(ν)**
**1120**	**C-O(ν)**
**840**	**C-H(γ)**

Figure S7. Raman and IR spectra of **7** (impurity G).

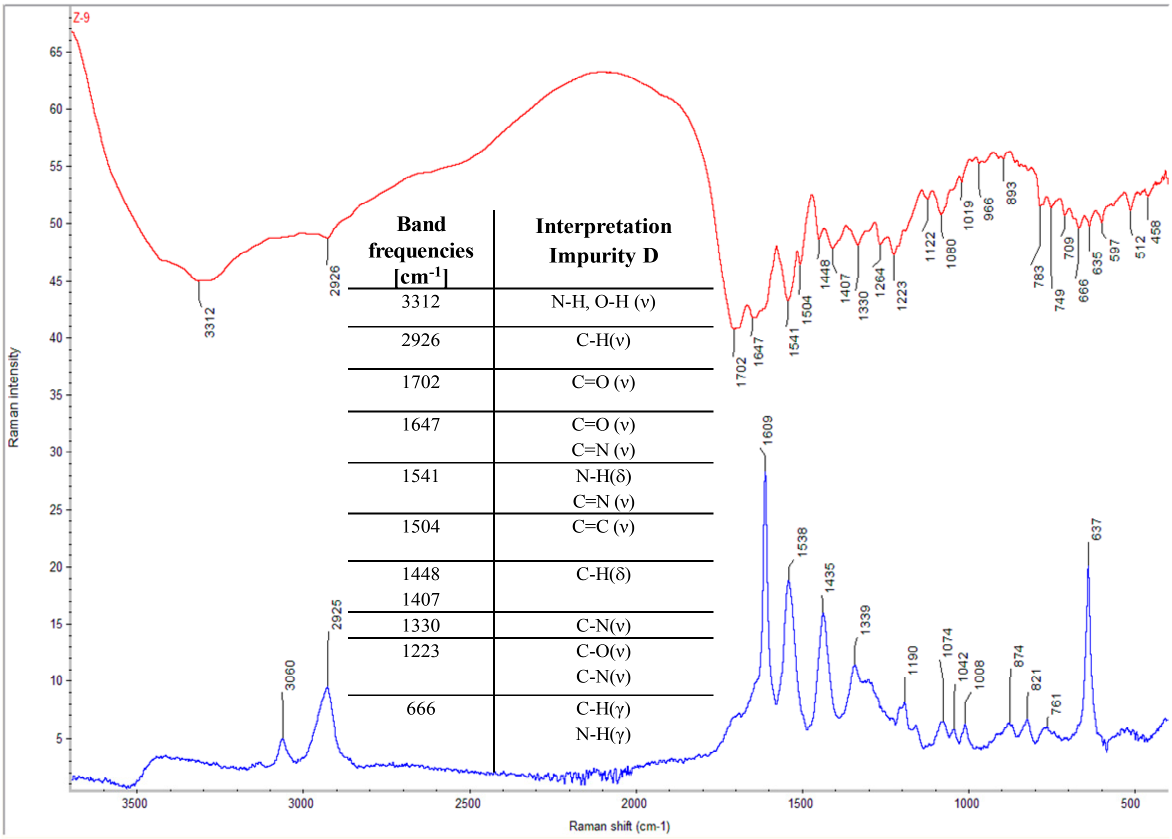

Band frequencies [cm-1]	Interpretation Impurity D
3312	N-H, O-H (ν)
2926	C-H(ν)
1702	C=O (ν)
1647	C=O (ν)<br>C=N (ν)
1541	N-H(δ)<br>C=N (ν)
1504	C=C (ν)
1448<br>1407	C-H(δ)
1330	C-N(ν)
1223	C-O(ν)<br>C-N(ν)
666	C-H(γ)<br>N-H(γ)

Figure S8. Raman and IR spectra of **8** (impurity D).

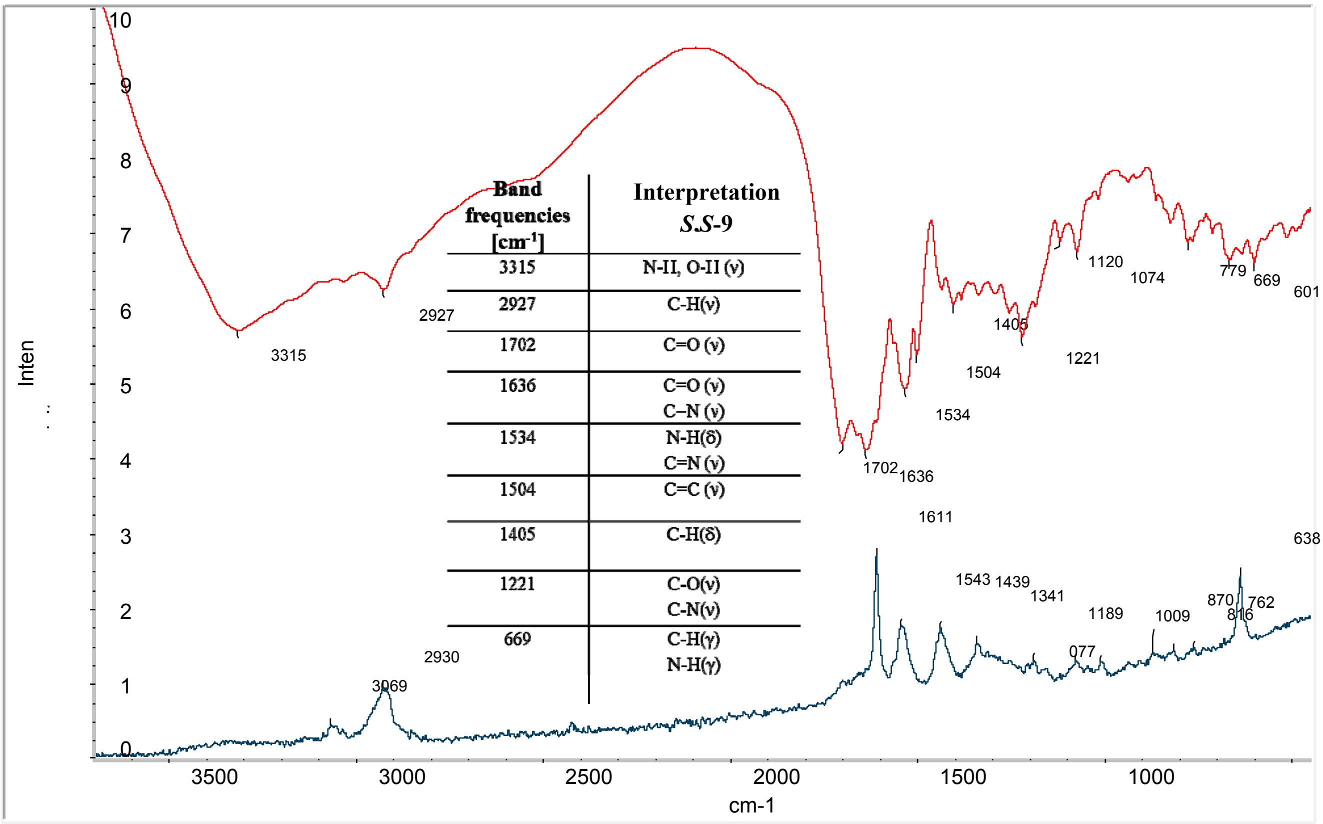

Band frequencies [cm-1]	Interpretation *S,S*-9
3315	N-II, O-II (ν)
2927	C-H(ν)
1702	C=O (ν)
1636	C=O (ν)<br>C–N (ν)
1534	N-H(δ)<br>C=N (ν)
1504	C=C (ν)
1405	C-H(δ)
1221	C-O(ν)<br>C-N(ν)
669	C-H(γ)<br>N-H(γ)

Figure S9. Raman and IR spectra of ***S,S*-9**.

Reference

1. Hanby, W.E.; Waley, S.G.; Watson, J. Synthetic polypeptides. Part II. Polyglutamic acid. *J. Chem. Soc.* **1950**, *632*, 3239–3249.
